# Plant Antimicrobial Peptides: Insights into Structure-Function Relationships for Practical Applications

**DOI:** 10.3390/cimb45040239

**Published:** 2023-04-21

**Authors:** Marina P. Slezina, Tatyana I. Odintsova

**Affiliations:** Vavilov Institute of General Genetics RAS, 119333 Moscow, Russia; omey@list.ru

**Keywords:** antimicrobial peptides (AMPs), antimicrobial activity, structure-function relationship, determinants of biological activity, novel anti-infective agents

## Abstract

Antimicrobial peptides (AMPs) are short polypeptide molecules produced by multicellular organisms that are involved in host defense and microbiome preservation. In recent years, AMPs have attracted attention as novel drug candidates. However, their successful use requires detailed knowledge of the mode of action and identification of the determinants of biological activity. In this review, we focused on structure-function relationships in the thionins, α-hairpinins, hevein-like peptides, and the unique Ib-AMP peptides isolated from *Impatiens balsamina*. We summarized the available data on the amino acid sequences and 3D structure of peptides, their biosynthesis, and their biological activity. Special attention was paid to the determination of residues that play a key role in the activity and the identification of the minimal active cores. We have shown that even subtle changes in amino acid sequences can affect the biological activity of AMPs, which opens up the possibility of creating molecules with improved properties, better therapeutic efficacy, and cheaper large-scale production.

## 1. Introduction

Antimicrobial peptides (AMPs) are ubiquitous short polypeptide molecules produced by multicellular organisms that are involved in host defense and microbiome preservation [1,2]. The widespread distribution of AMPs in evolutionary distant organisms highlights their crucial role in innate immunity [1,3,4]. Plant AMPs are extremely diverse, both structurally and functionally [5,6]. However, they share common features: they are usually short (10–90 amino acid residues), cationic (the net charge ranges from +2 to +11), disulfide-linked, enriched in hydrophobic residues (typically 50%), and amphiphilic. Cationic properties promote the binding of the peptides to the negatively charged cell walls and membranes of the pathogens. Further penetration into the membrane is made possible because of the amphiphilic properties of the peptide. The predominant mechanism of action of AMPs consists of membrane disruption and leakage of cytoplasmic constituents; however, some AMPs target intracellular processes and interfere with DNA and protein synthesis [7,8].

On the basis of 3D structural similarity, plant AMPs are grouped into several families [5]. Some families, such as defensins, are widely conserved in evolution. Others, such as thionins and α-hairpinins, are restricted to plants. There also exist AMPs present only in single plant species. Spectacular examples are AMPs from *Impatiens balsamina* and cysteine-free shepherdins from *Capsella bursa pastoris* [9,10]. Within species, AMPs are usually present in multiple variants, forming a unique pool of related molecules. It has been postulated that different isoforms result from functional divergence of AMP variants to increase the activity spectrum or obtain new functions [3].

Due to the development of multiple resistance to antibiotics, there is an urgent need for novel, highly efficient antimicrobials with a low incidence of resistance development [11]. The demand for new pesticides with reduced negative impacts on the environment for plant disease control is also increasing. AMPs have attracted attention as novel drug candidates due to their obvious advantages, such as rapid antimicrobial action, broad activity spectrum, synergism with antibiotics, and additional valuable (e.g., immunomodulatory) properties [12,13]. Another valuable benefit is that their modes of action are believed to be completely different from those of existing antibiotics, which results in slow resistance development. Furthermore, AMPs are considered safer than small-molecule drugs since their degradation produces natural amino acids and their half-life is short. In addition, they are usually less immunogenic than other polypeptide-based therapeutics [14,15].

The practical use of AMPs for the development of novel anti-infectives is often limited by insufficient knowledge of their mechanisms of action, which has so far only been studied for a limited number of peptides, and by the low yield of peptides that can be isolated from natural sources. Chemical synthesis and heterologous production are currently being used to enhance yield and decrease production costs. Another strategy involves the search for minimal structures that retain the antimicrobial activity of the original molecule. In cysteine-rich plant peptides, the γ-core, a structural signature with a GXCXnC motif adopting a β-hairpin (two β-strands connected by a loop) conformation, is claimed to be such a structure [16]. It was suggested that the γ-core represents an ancient membrane-interaction motif that determines the antimicrobial activity of cysteine-rich peptides. In our previous review, we summarized the available data on the γ-cores of plant AMPs and their antimicrobial properties [17]. However, sequence analysis of plant AMPs shows that not all of them harbor the γ-core motif. In this review, we focus on structure-function relationships in non-γ-core containing AMP families specific to the plant kingdom, such as thionins and hairpinins, and the unique peptides Ib-AMPs from *I. balsamina*. The available data on structure-function relationships in hevein-like AMPs, with a focus on regions beyond the γ-core motif, are also presented.

## 2. Thionins

### 2.1. General Characteristics

**Figure 1:**
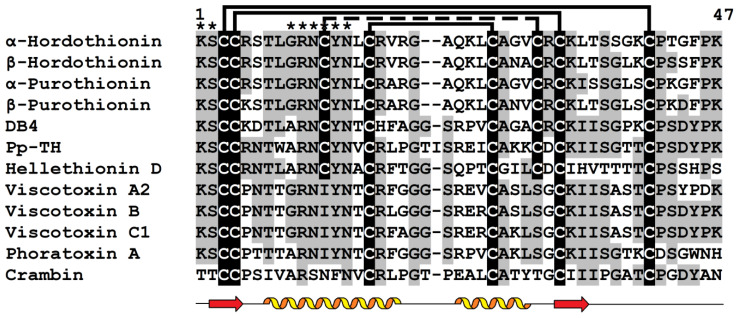
Multiple sequence alignment of selected thionins: α-hordothionin (GenBank AAA32966.1) and β-hordothionin (GenBank 1206255A) from *Hordeum vulgare*; α-purothionin (GenBank CAA65313.1) and β-purothionin (GenBank CAA65312.1) from *Triticum aestivum*, leaf-specific thionin DB4 from *H. vulgare* (UniProt P08772.2); Pp-TH from *Pyrularia pubera* (UniProt P07504.1); hellethionin D from *Helleborus purpurascens* (UniProt P60057.1); viscotoxin А2 (UniProt P32880.1), В (GenBank P08943.2), and С1 (UniProt P83554.1) from *Viscum album*; phoratoxin А from *California mistletoe* (UniProt P01539.1); crambin from *Crambe abyssinica* (UniProt P01542.2). Cysteine residues are highlighted in white on the black background. Conserved amino acid residues are highlighted in black on the grey background. Lines above the sequences denote disulfide bonds. Asterisks indicate conserved amino acid residues supposed to be involved in interactions with phospholipids. Secondary structure elements (α-heliсеs and β-strands) for β-purothionin (PDB 1BHP) are shown under the alignment as helices and arrows, respectively.

Thionins represent an AMP family found only in plants [18,19]. They are short (approximately 5 kDa) and mostly basic peptides containing six or eight cysteine residues (Figure 1). Thionins are separated into five structural classes. Class I thionins were discovered in the seeds of Poaceae plants [20]. They belong to the 8-Cys peptides and have a high positive charge on the molecules. Class II thionins occur in the leaves of barley (*Hordeum vulgare*) and the leaves and nuts of the parasitic plant *Pyrularia pubera* [21,22]. They also have eight cysteines, but they are less basic than class I thionins. Class III thionins were found in different mistletoe species [23,24]. They possess six cysteines, and the charge of their molecules is similar to that of class II thionins. Class IV thionins were isolated from *Crambe abyssinica* seeds [25]. They belong to 6-Cys peptides and are neutral. A wheat thionin, which is a truncated variant of class I thionins, belongs to class V [26]. Amino acid sequences of thionins belonging to different classes display high sequence similarity (Figure 1).

### 2.2. Biosynthesis

Thionins are synthesized as precursor proteins containing a signal peptide, the mature basic thionin domain, and an acidic C-terminal prodomain, which is supposed to be necessary for the transport of the mature thionin to vacuoles, cell walls, or protein bodies [19,27]. It also neutralizes the toxic properties of the mature thionin in the plant cells [28].

### 2.3. 3D Structure

**Figure 2:**
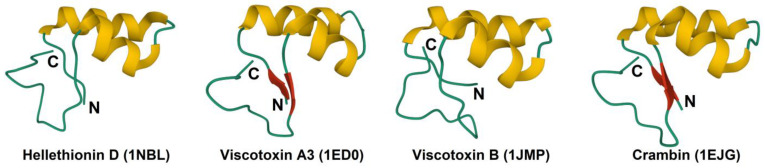
3D structure of plant thionins. The α-helices are colored yellow, and the β-structures are colored red. The N- and C-termini are indicated by N and C, respectively.

The amphipathic thionin molecule has the shape of the Greek letter Г, in which the long arm is formed by two antiparallel α-helices and the short one by two parallel β-strands (Figure 2). The C-terminal region of the thionin molecule forms a loop. The 3D structure is stabilized by three or four disulfide bonds. The groove between two structural domains is supposed to play a significant role in the biological activity of thionins [18,29]. Furthermore, Arg10 in the α1-helix is suggested to be essential for the structural stability of all thionins, as it is an abundant source of hydrogen bonds between β1, α1, and the C-terminal coil [30].

### 2.4. Biological Activity and Phospholipid Binding

Thionins display toxic properties toward different kinds of cells, including bacterial, fungal, and mammalian cancer cell lines. The molecular mechanisms underlying the toxic properties of thionins are not completely elucidated. However, it is generally acknowledged that thionins cause membrane lysis that triggers a series of destructive events in the cytoplasm that culminate in cell death [18]. Positively charged thionin molecules bind to patches of negatively charged phospholipids, such as phosphatidic acid or phosphatidylserine, and remove them from membranes. The conserved residues 1, 2, 9–14 that “cover” the groove between two structural domains of thionins are supposed to be involved in interaction with phospholipids (Figure 1). A nanopeptide corresponding to residues 7–15 of the *P. pubera* thionin sequence was synthesized, in which Cys12 was substituted with serine to increase stability. This short peptide was shown to bind to phosphatidylserine of the phospholipid membrane; however, its binding capacity was not as strong as that of the native thionin [31]. The formation of complexes with lipids solubilizes membranes and causes their lysis [18].

### 2.5. Structure-Function Relationships

The analysis of structure-function relationships in thionins started several decades ago. For wheat thionins (purothionins), it was shown that chemical modification of all amino groups, which significantly changes the charge of the molecule, leads to loss of toxicity for yeast and mouse cells (Table 1) [32]. Modification of only Tyr13 by nitration or iodination had the same effect. It was concluded that positively charged Lys residues are necessary for the preservation of structural integrity of the thionin molecule and interaction with the negatively charged cell surface and that the toxicity of the thionin directly depends on the tyrosine residue [32]. The importance of Tyr13 and Lys1 for the manifestation of toxic properties was confirmed by a sequence comparison of toxic thionins with non-toxic crambin, a thionin of *C. abyssinica* seeds, which showed that the residues Lys1 and Tyr13, which are conserved in toxic thionins, are substituted for Thr1 and Phe13 in the non-toxic crambin (Figure 1).

The importance of Tyr13 and Trp8 for biological activity was shown for the Pp-TH thionin from the parasitic plant *Pyrularia pubera*. This peptide is basic and has two tyrosine residues at positions 13 and 45, one tryptophan residue at position 8, and Asp32 instead of Arg present in most thionins (Figure 1). The Pp-TH displays a number of activities. It inhibits the growth of plant, bacterial and fungal pathogens, exhibits cytotoxic activity towards human and mouse tumor cell lines, and displays neurotoxic and hemolytic activities (Table 1) [34]. The mode of action of *Pyrularia* thionin involves membrane depolarization followed by Ca^2+^ influx. The Pp-TH activates phospholipase A2, which leads to disruption of membranes, hemolysis of erythrocytes, and eventually cell death. Prolonged iodination of *Pyrularia* thionin causes inhibition of all these cellular responses [34]. NMR studies showed that limited iodination modified Tyr45, which is more readily iodinated with the formation of the diiodo form than Tyr13 and Trp8. Limited iodination had virtually no effect on the thionin’s biological activity [40]. Conversely, prolonged iodination led to modifications of Trp8 and Tyr13. It was also demonstrated that modification of Trp8 with N-bromosuccinimide inhibited the hemolytic activity of the Pp-TH, showing that Trp8 is necessary for *Pyrularia* thionin activity [40].

The synthetic analogue of *Pyrularia* thionin, in which Asp32 was substituted with Arg32 that increased the charge of the molecule, had enhanced inhibitory activity against Gram-negative bacteria, while the activity against Gram-positive bacteria and fungi remained unchanged (Table 1) [35]. The overall structure of the mutant peptide was similar to that of the native peptide, except for a small decrease in helix content.

Based on the structure of Pp-TH, a 45% truncated peptide (residues from the 7th to the 32nd) was synthesized, consisting of two antiparallel α-helices stabilized by two disulfide bonds. The truncated peptide retained the antimicrobial activity and the mechanism of action of the intact Pp-TH (Table 1), thus it is the core region responsible for the antimicrobial activity of the thionin [36]. The misfolded PpTH(7-32)b with disulfide bridges C1-C2 and C3-C4 instead of C1-C4 and C2-C3 was completely inactive against Gram-negative bacteria and fungal pathogens and only slightly active against Gram-positive bacteria (Table 1). The PpTH(7-32)b did not adopt the antiparallel double helix conformation. However, at much higher concentrations, it was still able to suppress the growth of *C. michiganensis* (Table 1).

Vila-Perelló et al. synthesized the heterodimeric TH(7-19)(24-32R) peptide, in which the disulfide bonds connecting the two helical fragments were preserved (Table 1) [37]. Several other peptides derived from TH(7-19) and TH(24-32R) α-helical fragments, including linear and cyclic derivatives in which non-native disulfide bonds were incorporated, were also generated. The resultant 13- and 9-mer disulfide-linked peptides possessed enhanced antimicrobial activity compared to their linear counterparts, and their activity was comparable to that of the native thionin (Table 1).

Viscotoxins from *Viscum album* are thionins with antifungal activity and cytotoxic and anticancer properties towards human cells [41]. The antifungal activity of viscotoxin A3 was shown to be associated with channel formation in fungal membranes, leading to their disruption [42]. Cytotoxic properties to human cells were shown to be associated with membrane permeabilization, the production of reactive oxygen species, and apoptosis [43,44]. However, the hemolytic activity of viscotoxins is lower than that of other thionins, which is due to their lower hydrophobicity compared to other thionins [45]. Hydrophobicity was shown to be positively correlated with hemolytic activity [46].

Viscotoxins A2 (VA2), B (VB), and A3 (VA3) show high sequence similarity but differ in their cytotoxic properties against tumor cells. To elucidate the molecular bases of these differences, a comparative study of the interactions of three viscotoxins with model membranes was carried out (Table 1) [39]. The peptides differ in surface properties, which influence their interactions with membranes. The weaker hydrophobic character of VA2 compared to VA3 is believed to be responsible for its different affinity for membranes, resulting in lower cytotoxic activity. VB was shown to be much less active than VA2 and VA3, and it did not insert into model membranes. However, VB and VA2 differ in a very limited number of amino acid residues: Glu24Gln, Arg25Val, and Ser28Lys. The authors assume that a single Arg25 residue protruding from the hydrophobic plane formed by two α-helices, which are supposed to be involved in interactions with plasma membranes, is responsible for the different behavior of VB and, subsequently, for its lowest cytotoxicity towards tumor cells [39].

Sequence comparison complemented by 3D structure analysis of viscotoxins, which differ in cytotoxic activity against tumor cells, allowed Romagnoli et al. to predict amino acid residues associated with cytotoxic activity [47]. The importance of positively charged residues at positions 25 and 28 (Arg and Lys, respectively) and a negatively charged residue (Glu) at position 24 was deduced [47]. These residues (positions 24, 25, and 28) are located on the solvent-exposed side of the second helix and are supposed to be vital for interactions with membranes (Figure 1 and Figure 2).

### 2.6. Disulfide Bonds

The importance of intact disulfide bonds, an essential post-translational modification of cysteine-rich peptides, for the preservation of functions was shown for several thionins.

Molecular dynamics studies of *P. pubera* thionin demonstrated that disulfide bonds play a key role in the stabilization of its 3D structure and that the removal of only one disulfide bond was enough to significantly change the folding of the peptide. The same effect was achieved by improper Cys pairing, which was accompanied by a reduction or loss of activity [36,48]. The importance of disulfide bonds for the preservation of the 3D structure of thionins was also shown for hellethionin D, a thionin from the roots of *Helleborus purpurascens*. The reduction of disulfide bonds in this peptide led to its complete unfolding [49]. Studies of viscotoxins showed that nonreduced VA3 and VB bound with high affinity to phospholipid-containing membranes and preserved their structure when bound to membranes, while the reduced ones, on the contrary, formed aggregates. Furthermore, it was demonstrated that the native thionins were capable of disrupting membranes whereas the reduced proteins were not, pointing to the importance of disulfide bonds for thionin function [50].

## 3. Hevein-like Peptides

### 3.1. General Characteristics

**Figure 3:**
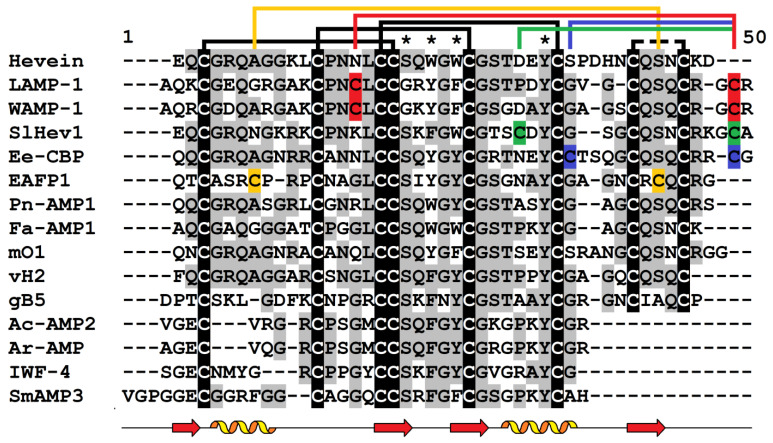
Multiple sequence alignment of hevein (UniProt P02877.2) and selected hevein-like AMPs: LAMP-1 (UniProt P86521.2) from *Leymus arenarius* and WAMP-1 (UniProt P85966.2) from *Triticum kiharae*; SlHev1 (GenBank UXX20393.1) from *Solanum lycopersicum*; Ee-CBP1 (GenBank AAP35269.1) from *Euonymus europaeus*; EAFP1 from *Eucommia ulmoides* (UniProt P83596.1); Pn-AMP1 from *Pharbitis nil* (UniProt P81591.1); Fa-AMP1 from *Fagopyrum esculentum* (UniProt P0DKH7.1); mO1 (UniProt A0A1S6EK91.1) from *Moringa oleifera*; vH2 from *Vaccaria hispanica* (PDB 5XDI); gB1 from *Ginkgo biloba* [53]; Ac-AMP2 from *Amaranthus caudatus* (GenBank AAB22102); Ar-AMP from *A. retroflexus* (UniProt Q5I2B2.1); IWF-4 from *Beta vulgaris* [55]; and SmAMP3 from *Stellaria media* (UniProt C0HJU5.1). Conserved cysteine residues are highlighted in white on a black background. The cysteine residues involved in the formation of the 5th disulfide bond are shown by red color for WAMP-1 and LAMP-1, by green color for SlHev1, and by blue color for Ee-CBP. Conserved amino acid residues are highlighted in black on the grey background. Lines above the sequences denote disulfide bonds. Asterisks indicate conserved amino acid residues of the chitin-binding site. Secondary structure elements (α- and 3_10_-heliсеs, and β-strands) for WAMP-1 (PDB 2LB7) are shown under the alignment as helices and arrows, respectively.

Hevein-like AMPs comprise an AMP family, whose members share structural similarity with hevein, an antimicrobial peptide isolated from the latex of *Hevea brasiliensis* [51]. Hevein-like AMPs consist of 30–45 amino acid residues and are enriched in glycine and cysteine residues involved in the formation of disulfide bridges [5,52]. Some peptides also have elevated ratios of other amino acids. For example, the hevein-like peptide gB1 from *Ginkgo biloba* leaves is enriched in proline residues [53], while the peptide vH1 from *Vaccaria hispanica* is glutamine-rich [54]. Most hevein-like peptides contain six or eight cysteine residues, and only a few possess 10 cysteines. The position of the 5th disulfide bridge in 10-Cys-containing hevein-like peptides is different (Figure 3), while the remaining cysteines are arranged in the motif found in hevein and other 8-Cys hevein-like peptides. The 6-Cys-containing hevein-like peptides are the truncated variants of the 8-Cys peptides and lack the fourth disulfide bond between the two last cysteines (Figure 3).

### 3.2. Biosynthesis

Hevein-like peptides are synthesized as precursor proteins containing a signal peptide, the mature peptide region, and a C-terminal domain, which can be short or long [53,54,55,56,57,58]. Some members of the family are encoded by multimodular precursors producing several hevein-like peptides [59,60,61]. Others originate from the post-translational proteolytic processing of class 1 chitinases or lectins [60,62].

### 3.3. 3D Structure

**Figure 4:**
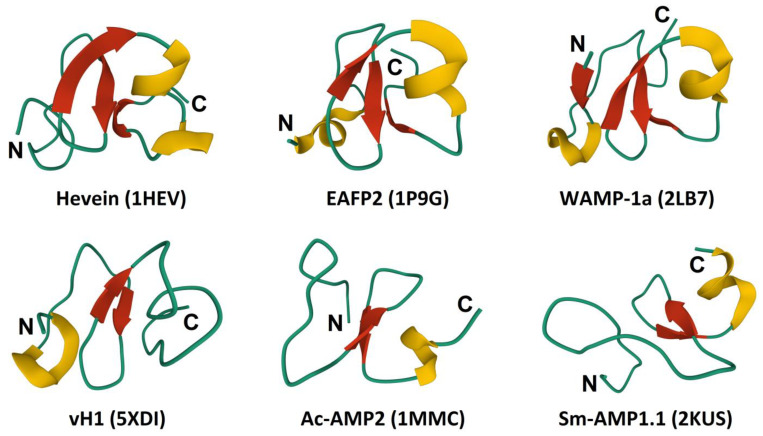
3D structure of hevein-like peptides. The helices are colored yellow, and the β-structures are colored red. The N- and C-termini are indicated by N and C, respectively.

The overall 3D structure of hevein-like AMPs includes a central β-sheet composed of two to four antiparallel β-strands and one or two short helical regions (Figure 4) [5,52]. Three disulfide bridges are strictly conserved. Of them, two adjacent disulfide bonds (C1-C4, C2-C5) are located perpendicular to each other, forming a knottin-like core. The most important feature is the presence of a chitin-binding site, which is stabilized by three conserved disulfide bonds and includes three aromatic amino acid residues and a serine residue (Figure 3). The chitin-binding site is found in other chitin-binding proteins involved in defense, such as class I and IV chitinases and lectins [63].

### 3.4. Biological Activity and Chitin-Binding

Hevein-like peptides display antifungal activity [5,52]. Some members of the family are active against Gram-positive and/or Gram-negative bacteria. The mode of action of hevein-like AMPs is poorly studied. The inhibitory activity against chitin-containing pathogens and against pathogens devoid of chitin suggests the existence of multiple mechanisms of action. Regarding the antifungal activity, the ability to interact with the chitin of fungal cell walls through the chitin-binding site is supposed to play a significant role in the mechanisms of fungal growth inhibition [64]. The binding of the hevein-like peptide to the chitin oligomers is supposed to interfere with fungal cell wall morphogenesis. However, beyond the cell wall, intracellular targets have also been shown [65]. Studies of the complexes between hevein and chitin (GlcNAc)_1–5_ oligosaccharides showed that the aromatic residues Trp21, Trp23, and Tyr30 of the chitin-binding site are involved in interactions with oligosaccharides through van-der-Waals and CH-π interactions [66,67,68]. These interactions are stabilized by a hydrogen bond between Ser19 and the acetamide moiety of a GlcNAc residue.

### 3.5. Structure-Function Relationships

Several studies explored the role of mutations in the chitin-binding site on the capacity of hevein to bind chitin oligosaccharides. A hevein mutant Hev32 harboring a C-terminal truncation of 10 amino acid residues showed no significant loss in binding affinity [69] and thus was named the minimum hevein domain necessary for oligosaccharide binding. The mutation of Ser19 to Asp in the hevein chitin-binding site resulted in a considerable decrease in the binding of the mutant peptide to (GlcNAc)_3_ due to the loss of a hydrogen bond between Ser19 and the carbonyl of the acetamide group [70]. Thus, the importance of this hydrogen bond for efficient binding of oligosaccharides was demonstrated. Studies of pseudohevein, a natural mutant of hevein with a substitution of Trp21 for Tyr, showed no significant differences in oligosaccharide binding capacity between the two peptides [67]. The importance of the interactions between the hydrophobic CH groups of carbohydrate residues and the π-electron systems of aromatic amino acid residues in ligand recognition in carbohydrate-binding proteins was emphasized by Muraki et al. [68]. Replacement of each of the conserved aromatic residues of the chitin-binding site in 6-Cys containing hevein-like peptide Ac-AMP2 with alanine resulted in a decrease in binding affinity to chitin, pointing to the importance of all three aromatic residues for efficient ligand binding [71]. Substitution of Phe in the chitin-binding site of Ac-AMP2 with non-natural amino acid residues with larger aromatic rings resulted in increased affinity, while the mutation of Tyr20 to Trp decreased the mutant peptide’s affinity to chitin [71].

Similar to other plant AMP families, studies of the molecular diversity of hevein-like peptides have shown that often more than one peptide is found in a single plant species. These peptide variants are usually very similar in amino acid sequences, which opens up opportunities to study the role of minor sequence variations in biological activity. Here are some examples.

Two 6-Cys hevein-like peptides, Ac-AMP1 and Ac-AMP2, were purified from the seeds of *Amaranthus caudatus* (Figure 3, Table 2). Ac-AMP1 consists of 29 amino acid residues, and Ac-AMP2 is composed of 30 residues. Their amino acid sequences are identical except for an additional C-terminal Arg in Ac-AMP2, which increases its net charge. The NMR studies of Ac-AMP2 showed that its main structural element is a twisted antiparallel β-sheet composed of two strands, Met13 to Ser16, and Tyr20 to Lys23, and a short helical segment, Pro25 to Gly29 (Figure 4) [72]. Antimicrobial assays demonstrated that the peptides Ac-AMP1 and Ac-AMP2 inhibited the growth of six species of plant pathogenic fungi (*Alternaria brassicola*, *Ascochyta pisi*, *Botrytis cinerea*, *Colletotrichum lindemuthianum*, *Fusarium culmorum,* and *Verticillium dahlia*), a saprophyte, *Trichoderma hamatum,* and the Gram-positive bacteria *Bacillus megaterium* and *Sarcina lutea* (Table 2). The antifungal activity against some pathogens (*A. pisi*, *B. cinerea*, *C. lindemuthianum*, *F. culmorum,* and *V. dahlia*) was the same for both peptides, while Ac-AMP2 appeared much more effective against *A. brassicola* and *T. hamatum*. The antibacterial activity against the tested Gram-positive bacteria was higher for Ac-AMP2. Thus, the C-terminal Arg in Ac-AMP2 increased the antimicrobial activity against some fungal pathogens and Gram-positive bacteria.

Two 8-Cys hevein-like peptides designated Fa-AMP1 and Fa-AMP2 were isolated from the seeds of *Fagopyrum esculentum* [82]. Both peptides consist of 40 amino acid residues. Their amino acid sequences are identical except for the C-terminal residue: in Fa-AMP1, it is Lys, and in Fa-AMP2, it is Arg. Despite the fact that the variable residue is basic in both peptides, the antimicrobial activity against the tested seven species of fungi and bacteria differed (Table 2). The tested microbes included two fungal species, *F. oxysporum* and *Geotrichum candidum*, three species of Gram-negative bacteria (*Erwinia carotovora, Agrobacterium rhizogenes,* and *A. radiobacter*), and two species of Gram-positive bacteria (*Clavibacter michiganensis* and *Curtobacterium floccumfaciens*). Fa-AMP1 was much more active against *F. oxysporum* than Fa-AMP2. On the contrary, Fa-AMP2 was more potent against *G. candidum* and *A. radiobacter*. The activity against the remaining microorganisms was similar for both peptides.

Two hevein-like peptides named Pn-AMP1 and Pn-AMP2 were isolated from the seeds of *Pharbitis nil* [81]. They contained 41 and 40 amino acid residues, respectively, including eight cysteines. The peptides had identical amino acid sequences except for an additional C-terminal Ser in Pn-AMP1. Pn-AMPs displayed potent antifungal activity against nine species of fungi and oomycetes (*B. cinerea*, *C*. *langenarium*, *Sclerotinia sclerotiorum*, *F. oxysporum*, *Rhizoctonia solani*, *Phytophtora capsici*, *Phytophtora parasitica*, *Phythium* spp., and *Saccharomyces cerevisiae*) (Table 2). Pn-AMP2 was much more active than Pn-AMP1 on most pathogens except *R. solani*. The activity of both peptides against *P. parasitica* was similar. Studies of the mode of action of Pn-AMP1 showed that the peptide penetrated into the hyphae of *B. cinerea* and *P. parasitica* and localized at the septum and hyphal tips, which triggered bursts of hyphal tips and leakage of the cytoplasmic constituents. In yeasts, Pn-AMP1 caused actin depolarization [65].

Two highly similar 10-Cys hevein-like peptides, EAFP1 and EAFP2, were isolated from the bark of *Eucommia ulmoides* [80]. Both peptides consist of 41 amino acid residues and have pyroglutamic acid at the N-terminus. Their molecules are stabilized by five disulfide bridges: C1-C5, C2-C9, C3-C6, C4-C7, and C8-C10. The 5th disulfide bond, C2-C9, is unique since it brings together the N- and C-terminal halves of the molecule, producing a cationic cluster of four arginine residues [86,87]. EAFP2 adopts a compact conformation consisting of a 3_10_-helix (Cys3-Arg6), an α-helix (Gly26-Cys30), and a three-stranded antiparallel β-sheet (Cys16-Ser18, Tyr22-Gly24, and Arg36-Cys37) (Figure 4). The peptides exhibit antifungal activity against a variety of fungi, such as *Aculops lycopersici*, *V. dahliae*, *F. oxysporum*, *F. moniliforme*, *Colletotrichum gossypii*, and the oomycete *P. infestans*. However, they are inactive against the Gram-positive (*Pseudomonas syringae*) and Gram-negative (*B. megaterium*) bacteria (Table 2). Sequence comparison of both peptides shows that EAFP1 and EAFP2 differ by a single amino acid residue at position 27: a polar Asn in EAFP1 is substituted with a hydrophobic residue Ala in EAFP2. This residue is located in the second α-helix formed by residues 26–30 and is part of the hydrophobic cluster in the amphiphilic structure of the peptide. This replacement affects the antifungal activity of the peptides. EAFP2 is more potent against *A. lycopersici* and *F. moliforme*, while EAFP1 is more efficient against *F. oxysporum* and *C. gossypii* (Table 2).

The role of particular amino acid residues and regions of the molecule in the antimicrobial activity of hevein-like peptides was studied in more detail for the 10-Cys-containing hevein-like peptides named WAMPs isolated from the wheat species *Triticum kiharae* [75]. WAMPs possess potent antimicrobial activity against chitin-containing and chitin-free pathogens. The solution structure of WAMP-1a showed that its molecule contains an antiparallel, four-stranded β-sheet, a 3_10_-helix, and an α-helix (Figure 4) [88]. A conserved serine residue in the chitin-binding site of WAMPs is replaced by a glycine residue that reduces the carbohydrate-binding capacity of the peptide [70], but increases its amphiphilicity (Figure 4) [88]. Homologous peptides were discovered in related Poaceae species [57,74,76,89]. Their amino acid sequences are highly conserved except for a variable position 34, which is located in the solvent-exposed loop connecting the α-helix (residues 29–32) with the β4 strand (residues 36–39). Several residues are found at this position: Lys, Ala, Asn, Glu, and Val. Studies of the antifungal activity of WAMPs showed variation in the degree of inhibition of phytopathogenic fungi depending on the fungal species (Table 2) [78]. All tested WAMPs exhibited potent antifungal activity against *Bipolaris sorokiniana*; WAMP-3.1 (E34) and WAMP-1b (A34) were the most active, while WAMP-2 (K34) showed the weakest activity. The activity of WAMPs against *F. oxysporum* was lower compared to *B. sorokiniana*. The antifungal activity against this fungus decreased in the following order: WAMP-3.1 > WAMP-2 > WAMP-4 > WAMP-5 > WAMP-1b. All tested peptides inhibited *Alternaria alternata* spore germination; however, the degree of inhibition was lower than that of *F. oxysporum* and *B. sorokiniana*. *Cladosporium cucumerinum* was efficiently suppressed by WAMP-2. WAMP-4 and WAMP-5 were also efficient inhibitors of this fungus, while WAMP-1b and WAMP-3.1 failed to inhibit *C. cucumerinum*.

The molecular mechanisms underlying the antifungal activity of WAMPs were studied against *Fusarium* pathogens. It was found that WAMPs act as specific inhibitors of fungalysin, a secreted Zn-metalloproteinase of *Fusarium* fungi that targets plants defense chitinases and acts as the pathogen’s effector [77]. The ability to inhibit the metalloproteinase is obviously associated with WAMPs’ structural similarity with the chitin-binding domain of plant class I chitinases. The homologues differing in position 34 differed in the degree of proteinase inhibition. WAMP-1b and WAMP-2 appeared to be effective inhibitors of fungalysin, while WAMPs 3.1 and 4 were not. Yet the latter WAMPs preserved their ability to inhibit the growth of fungal pathogens in vitro, as shown above, suggesting the existence of an alternative fungalysin-independent mechanism of action. To gain insight into the underlying mechanisms, we studied the antifungal activity of synthetic peptides derived from the central (WAMP-G1 and WAMP-G2), N-, and C-terminal regions (WAMP-N and WAMP-C, respectively) of one of the WAMPs, namely WAMP-2 [78]. The sequence of WAMP-G2 possessed all three aromatic residues of the chitin-binding site, which are involved in binding carbohydrates (Tyr22, Phe24, and Tyr31 in WAMP-2). WAMP-G1 was shorter than WAMP-G2 by four amino acid residues and thus lacked the last Tyr residue of the chitin-binding site. In the WAMP-2 molecule, WAMP-N, WAMP-G1/G2, and WAMP-C peptide regions occupy adjacent clusters on the surface of the molecule. The antifungal activity of WAMP-2-derived peptides was assayed against seven plant pathogenic fungi causing harmful diseases in crops. WAMP-C was the most active peptide against *C. cucumerinum*. It was much more active than the intact WAMP-2 molecule (Table 2). This indicates that the activity of WAMP-2 against *C. cucumerinum* is not connected with its chitin-binding site but depends on the C-terminal region of the molecule. WAMP-C has the highest positive charge (+3) of all WAMP-2-derived peptides, suggesting the strongest electrostatic interactions with negatively charged groups of fungal cell walls and/or membranes. The peptide is predicted to possess an α-helical region [78]. The surface of WAMP-C is mostly hydrophilic; thus, the formation of pores and insertion into the fungal membranes seem unlikely. Of all peptides, WAMP-N was the most active against all fungi except for *C. cucumerinum* (Table 2). This points to the important role of the N-terminal region of the WAMP-2 molecule in antifungal activity. The WAMP-N is predicted to be α-helical and amphiphilic [78], therefore, the penetration through fungal membranes as a mechanism of action seems possible. WAMP-G2 was much more active than WAMP-G1, since it inhibited the spore germination of all fungi. Given that WAMP-G2 differed from WAMP-G1 by four amino acid residues from the C-terminus, including Tyr31 of the chitin-binding site, the discovery that WAMP-G2 was much more active than WAMP-G1 allowed us to hypothesize that all three conserved aromatic residues of the chitin-binding site are essential for the antifungal activity of WAMP-G2.

In summary, the results obtained show that in addition to fungalysin inhibition, WAMPs possess a fungalysin-independent antifungal mechanism.

## 4. α-Hairpinins

### 4.1. General Characteristics

**Figure 5:**
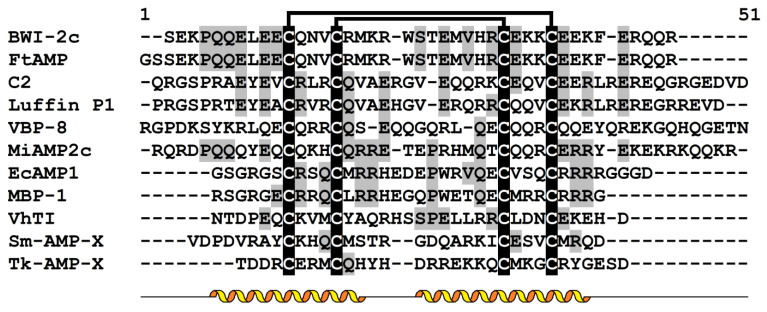
Multiple sequence alignment of α-hairpinins: BWI-2c (UniProt P86794) from *Fagopyrum esculentum*; FtAMP from *F. tataricum* [94]; C2 (UniProt Q9ZWI3) from *Cucurbita maxima*; luffin P1 (UniProt P56568) from *Luffa aegyptiaca*; VBP-8 (PDB 6O3Q) from *Solanum lycopersicum*; VhTI (UniProt P85981.1) from *Veronica hederifolia*; MiAMP2c from *Macadamia integrifolia* [95]; EcAMP1 from *Echinochloa crus-galli* (PDB 2L2R); MBP-1 from *Zea mays* (UniProt P28794.1); Sm-AMP-X from *Stellaria media* (UniProt U4N938.1); and Tk-AMP-X from *T. kiharae* [96]. Cysteine residues are highlighted in white on the black background. Conserved amino acid residues are highlighted in black on the grey background. Lines above sequences denote disulfide bonds. Secondary structure elements (α-heliсеs) are shown for BWI-2c (PDB 2LQX) as helices under the alignment.

**Figure 6:**
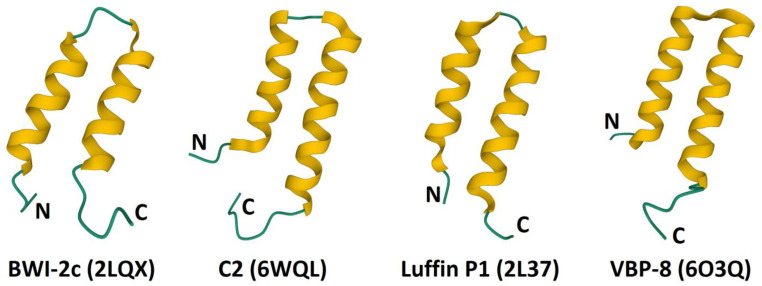
3D structure of α-hairpinins. The α-helices are colored yellow. The N- and C-termini are indicated by N and C, respectively.

The α-hairpinins are short (less than 50 amino acid residues) AMPs with a 4-Cys motif C_1_X_3_C_2_X_n_C_3_X_3_C_4_ and disulfide connectivities C1-C4 and C2-C3. They were discovered in a number of plant species (Figure 5) [90,91,92,93,94,95,96]. Although the sequence similarity between hairpinins of different species is rather low, their three-dimensional structure is similar and resembles a hairpin, in which two antiparallel α-helical regions connected by a loop are brought together by two disulfide bridges (Figure 6) [90,91,92,93,97]. The N- and C-terminal “tails” are unstructured. The same helix-loop-helix structural motif is found in thionins (see above) and some animal toxins [97].

### 4.2. Biosynthesis

The α-hairpinins are synthesized as precursor proteins of two types. The first type of precursor protein consists of a signal peptide, a Cys-rich domain with 2–4 α-hairpinin motifs, and a hydrophobic C-terminal domain showing sequence similarity to the seed storage proteins vicilins [95,98]. In the second type of precursors, a signal peptide is followed by a multidomain region consisting of 5–12 hairpinin modules and a short C-terminal prodomain, which has no sequence similarity to known proteins [96,99].

### 4.3. Biological Activity

The α-hairpinins display a variety of functions, including antifungal, antibacterial, trypsin inhibition, and ribosome-inactivating [90,91,92,93,94,95,96,97,98,99,100,101]. The mode of antimicrobial action has been studied only for selected peptides and remains poorly understood. Sm-AMP-X from *Stellaria media* and EcAMP1 from *Echinochloa crus-galli* inhibited elongation of hyphae without membrane disruption [91,99]. Ec-AMP1 was shown to bind to the surface of fungal conidia and then accumulate in the cytoplasm, avoiding the disturbance of membrane integrity. For Tk-AMP-X2 from *T. kiharae*, the membrane disruptive mechanism was also excluded since the surface of the peptide is entirely hydrophilic [97]. Studies of the morphology of *E. coli* cells treated with MBP-1 (maize basic peptide 1) also showed that membrane integrity was not disturbed, suggesting an intracellular mechanism of antibacterial action mediated by DNA binding and followed by inhibition of DNA synthesis [100].

### 4.4. Structure-Function Relationships

Structure-function relationships were studied for antifungal hairpinins and trypsin inhibitors. The maize α-hairpinin named MBP-1 inhibited the growth of bacteria (*E. coli* and *C. michiganense*) and fungi belonging to the genera *Fusarium*, *Alternaria*, *Sclerotinia,* and *Aspergillus* [101]. To study the molecular determinants responsible for the antibacterial activity of MBP-1, two peptide variants were synthesized (Table 3) [100]. In the first variant, the Trp20 residue located in the loop connecting two α-helices was replaced by alanine. In the second variant, all cysteine residues were substituted with alanine. Antibacterial assays with *E. coli* DH5-α showed that the chemically synthesized MBP-1 was active, while both mutant variants were inactive at concentrations below 400 μM, pointing to the importance of Trp20 and disulfide bridges for the antibacterial activity (Table 3). Molecular modeling demonstrated that removing the disulfide bridges from MBP-1 produced dramatic structural changes in the peptide, which could result in a loss of activity due to decreased DNA binding [100].

The importance of N- and C-terminal regions for the antifungal activity of hairpinins was shown for Sm-AMP-X, a hairpinin from *S. media* seeds [99]. The Sm-AMP-X peptide is active against fungi, such as *F. oxysporum*, *F. solani*, *Aspergillus niger*, *Alternaria alternata,* and *B. cinerea*, but is inactive against bacteria (Table 3) [99]. Two truncated peptides were synthesized, one of which, named Sm-AMP-X1, corresponded to the sequence between the 1st and 4th cysteine residues with both disulfides preserved, and the second peptide variant, named Sm-AMP-X2, had the sequence between the 2nd and 3rd cysteines of the intact hairpinin. It was found that the antifungal activity of both truncated peptides was lower than that of the intact peptide, with the lowest activity for the shortest peptide (Table 3). This correlated with a progressive decrease in α-helical content: Sm-AMP-X > Sm-AMP-X1 > Sm-AMP-X2. Thus, it was suggested that the “tails” of the molecule contribute to the antifungal activity of Sm-AMP-X either through direct interaction with the fungi or through stabilization of the helical structure [99]. Similar results were obtained with the truncated variants of EcAMP1 [103]. Furthermore, the modified variants EcAMP1-X3, with a single Trp20Ala substitution, and EcAMP1-Hyp, with a Pro19Hyp substitution in the second α-helical region of the molecule, were shown to be less active towards *Fusarium* fungi than the original molecule [102,103]. Molecular dynamics simulations indicated that Pro19 is important for binding carbohydrates located in the cell walls of spores.

A possibility was explored to modify the structure of hairpinins to obtain novel functions. A hairpinin Tk-AMP-X2 was used as a model [97]. As mentioned above, some animal toxins possess the same α-helical hairpin fold and the same cysteine pattern as plant α-hairpinins, among them κ-hefutoxin 1 from the venom of the scorpion *Heterometrus fulvipes*, which displays potassium channel blocking activity [104]. To obtain potassium channel-blocking function on the Tk-AMP-X2 hairpin scaffold, the Tyr and Lys pair essential for potassium channel blocking activity was introduced into the Tk-AMP-X2 molecule instead of Glu6 and Met22 to produce the mutant molecule Tk-hefu. An additional substitution (Lys23Glu) was made to avoid the positive charge at this position that might decrease the activity. Electrophysiological studies convincingly showed that, in contrast to the native Tk-AMP-X2, the mutant peptide Tk-hefu acquired the ability to block potassium channels (Table 3). Thus, it was concluded that α-hairpinins can serve as structural templates for designing molecules with novel properties [97].

The role of different amino acid residues in protease inhibition was studied on the α-hairpinin VhTI isolated from seeds of *Veronica hederifolia*, which displayed the activity of a trypsin inhibitor (Table 3) [90]. The 3D structure of the peptide in complex with trypsin was solved by X-ray crystallography, and the residues involved were identified. A synthetic, truncated form of VhTI consisting of residues 5–31 and containing both helices but lacking the unstructured “tails” (residues 1–4 and 32–34) was prepared (Table 3). It was shown that the loop connecting both helices in the truncated peptide inserts into the active site of the enzyme and that only the core 27-amino acid segment of the peptide is required for full inhibitory activity of VhTI. Analysis of the 3D structure of the trypsin-inhibitor complex also showed that Met10, Ala13, Gln14, and Arg15 residues of the VhTI blocked the active site of the protein and completely prevented substrate from binding. Arg15 of VhTI, located in the substrate specificity pocket of trypsin, plays a key role in interactions with trypsin: the Arg15 side chain forms a salt bridge with the side chain of trypsin Asp189 and hydrogen bonds with Ser190 and Gly219.

The residues vital for trypsin inhibition were further studied using a hairpinin called FtAMP from tartary buckwheat seeds obtained by gene cloning and expression in *E. coli* cells [94]. The peptide was bifunctional: it displayed trypsin-inhibitory activity and antifungal properties. To study structure-function relationships, two mutant variants, FtAMP-R21A and FtAMP-R21F, were produced by site-directed mutagenesis (Table 3). Both mutant peptides lost trypsin-inhibitory activity. However, FtAMP-R21A and FtAMP-R21F peptides became active against elastase and α-chymotrypsin, respectively (Table 3). It was concluded that Arg21 in the inhibitory site loop of FtAMP is necessary for trypsin inhibition. Antifungal assays showed that all three peptides exhibited strong antifungal activity against several fungi, such as *F. oxysporum*, *Trichoderma koningii*, and *Rhizopus* sp. Thus, mutations in the FtAMP inhibitory site had no effect on the antifungal properties of the peptides. It was hypothesized that the antifungal activity is associated with α-helical regions. It still remains unclear whether the helices carry antifungal determinants or are crucial for α-helix formation, which in turn is necessary for the manifestation of antimicrobial properties.

## 5. *Impatiens balsamina* Antimicrobial Peptides (Ib-AMPs)

### 5.1. General Characteristics

**Figure 7:**
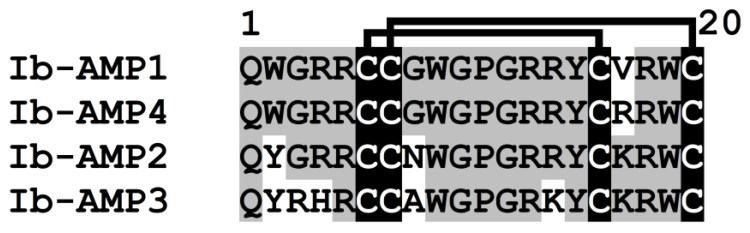
Multiple sequence alignment of the antimicrobial peptides Ib-AMPs from *Impatiens balsamina* [9]. Cysteine residues are highlighted in white on the black background. Conserved amino acid residues are highlighted in black on the grey background. Lines above the sequences denote disulfide bonds.

From the seeds of *I. balsamina*, four short (20 amino acid residue) peptides named Ib-AMP1-4 were isolated [9]. The peptides were highly basic (5–6 arginines) and possessed four cysteine residues arranged in the motif CCX_8_CX_3_C (Figure 7). Two disulfide bridges were formed between C1 and C3 and between C2 and C4 [105]. The peptides were highly similar in amino acid sequences. The sequence identity between Ib-AMP1 and Ib-AMP2 and between Ib-AMP2 and Ib-AMP3 was 85% (three amino acid substitutions), while the sequence identity between Ib-AMP1 and Ib-AMP4 amounted to 95% (one amino acid replacement).

### 5.2. Biosynthesis

The Ib-AMP1-4 peptides are produced during proteolytic processing of a single transcript. The precursor protein contains a signal peptide and six mature peptide domains (Ib-AMP1 is repeated three times, while other Ib-AMPs only once) separated by acidic propeptide regions ranging from 16 to 35 residues in length [9].

### 5.3. 3D Structure

The solution structure of Ib-AMP1 was solved by NMR spectroscopy [105]. The peptide (residues 6–20) adopts a loop structure devoid of α-helices and β-structure, which is stabilized by disulfide bonds, while the peptide without disulfide bonds due to the substitutions of cysteines with α-aminobutyric acid adopts a random coil conformation [105]. The Ib-AMP1 peptide has two hydrophilic patches at opposite ends of the molecule consisting of residues Arg4, Arg5, and Arg18 and residues Arg13 and Arg14, respectively, which are separated by a hydrophobic patch consisting of residues Trp9, Val17, and Trp19 and the cysteines. The peptide does not form an amphipathic helix [106].

### 5.4. Biological Activity

IbAMPs inhibit the growth of a wide range of plant pathogenic fungi and bacteria, but do not lyse human erythrocytes and have no effect on the fibroblast membranes [9]. For Ib-AMP1, it was shown that it binds to the fungal cell surface or penetrates into fungal cell membranes [106]. Since the peptide does not form an amphipathic helix, it was suggested that it does not act through nonspecific membrane disruption [106]. Studies of the bactericidal effect of Ib-AMP1 on *Staphylococcus aureus* showed that the peptide did not induce depolarization of the cytoplasmic membranes [107]. It was speculated that Ib-AMP1 targets intracellular components of bacterial cells.

IbAMP4 was also shown to be active against human bacterial pathogens, including multidrug-resistant strains [108,109,110]. The peptide displayed bactericidal activity but showed no cytotoxic or hemolytic activity towards human cells up to 100 mM concentration [108,111]. IbAMP4 acted in synergy with conventional antibiotics, such as vancomycin or oxacillin, against *Enterococcus faecalis* [109].

### 5.5. Structure-Function Relationships

The Ib-AMP4 peptide differs from Ib-AMP1 by a single amino acid replacement of Val17 in Ib-AMP1 with arginine in Ib-AMP4. Homology molecular modeling showed that Arg17 increases the hydrophilic region formed by residues Arg4, Arg5, and Arg18. This substitution is accompanied by an increase in the antifungal activity of Ib-AMP4 against the fungus *B. cinerea* and the Gram-positive bacteria (Table 4) [9]. Conversely, the activity against the fungus *Verticillium alboatrum* was higher for Ib-AMP1.

The reduction of disulfide bonds in Ib-AMP1 led to a fourfold decrease in its antifungal activity against *Aspergillus flavus* and *Candida albicans* (Table 4) [106]. This result points to the essential role of disulfide bonds in the antifungal activity of Ib-AMP1 [106]. In another study, four linear variants of Ib-AMP1 and Ib-AMP4 were synthesized; in two of them, all four cysteine residues were substituted with L-α-aminobutyric acid; in two other variants, all L-amino acids were replaced by D-amino acids, and cysteines were substituted with D-α-aminobutyric acid (Table 4) [111]. In the second mutant series, to understand the role of Arg and Trp residues in the antifungal activity of Ib-AMP4, the linear peptide derivatives with L-α-aminobutyric acid instead of cysteines, in which each amino acid was consecutively substituted with Arg or Trp, were synthesized (Table 4) [111]. The activity of all Ib-AMP variants against fungal and yeast strains was assayed. The linear Ib-AMP1 and Ib-AMP4 derivatives (with L-α-aminobutyric acid instead of cysteines) were as active as the native peptides against *B. cinerea*, despite the fact that the linear peptides adopted a random coil conformation instead of a loop conformation. However, they were less active against the fungi *Neurospora crassa*, *F. culmorum*, and *S. cerevisiae*. The activity of linear Ib-AMP variants against *Pichia pastoris* was even higher than that of the native peptides. Both Ib-AMP derivatives with all D-amino acids were 2–6 times more active than those with all L-amino acids. The introduction of R or W residues in the Ib-AMP4 sequence did not influence the antifungal activity of the peptide derivatives by more than twofold as compared to the non-modified Ib-AMP4 (Table 4).

In order to study the role of disulfide bonds in the antibacterial activity of Ib-AMP1, its linear analogues with L-Pro, D-Pro, or peptoid residues (Nala and Nlys) at the central position of the molecule were synthesized [107]. The antibacterial activity of the analogues increased by a factor of 3.7–4.8 compared to the native Ib-AMP1, providing evidence that disulfide bonds are not significant for its antibacterial potency (Table 4). Studies of the mode of action of Ib-AMP1 analogues showed that, in contrast to Ib-AMP1, all linear analogs displayed bactericidal effects on *S. aureus* cells through complete depolarization of the membrane potential without membrane disruption and the formation of small channels leading to leakage of ions or protons [107].

## 6. Conclusions

Antimicrobial peptides are key effector molecules of the plant’s innate immunity and are emerging as promising alternatives to conventional antibiotics that are less prone to resistance development [1]. In the present review, we highlighted structure-function relationships in plant AMPs that do not possess a typical γ-core signature but still exhibit potent antimicrobial activity, such as the thionins, α-hairpinins, and *I. balsamina* Ib-AMPs. We also described structure-function studies of an enigmatic hevein-like AMP family with a special focus on the role in activity of the molecule parts beyond the γ-core signature. These studies contribute to the elucidation of the mode of action of thionins, α-hairpinins, hevein-like peptides, and Ib-AMPs and evaluate their potential for the development of novel anti-infective drugs that ultimately selectively kill the pathogen but preserve the host’s microbiome intact.

We showed that even subtle changes in amino acid sequences can affect the antimicrobial properties of AMPs. In the thionin family, single amino acid changes (Lys1Thr and Tyr13Phe) turned toxic thionins into the non-toxic crambin. The substitution of Asp32 with Arg in *P. pubera* thionin Pp-TH increased activity [35], while modification of Trp8 and Tyr13 in Pp-TH and Tyr13 in wheat thionins had an opposite effect [32,40]. For viscotoxins, the importance of positively charged residues at positions 25 and 28 and a negatively charged residue at position 24 for cytotoxicity was emphasized [39]. In the hevein-like AMPs, the addition of a C-terminal Arg in Ac-AMP2 compared to Ac-AMP1 increased activity against Gram-positive bacteria [83]. The substitution of a polar Asn in EAFP1 with a hydrophobic Ala in EAFP2 changed activity against different fungi [80]. Even the substitution of one positively charged residue with another (Lys in Fa-AMP1 with Arg in Fa-AMP2) at the C-terminus affected antimicrobial properties [82]. In the hevein-like peptides WAMPs, the residues at position 34 influenced the efficiency of fungalysin inhibition and suppression of fungal growth [77,78]. In Ib-AMPs, the substitution of Val at position 17 in Ib-AMP1 with Arg in Ib-AMP4 increased activity against Gram-positive bacteria [9]. Thus, an increase in charge is usually accompanied by enhanced activity against Gram-positive bacteria. In the α-hairpinin AMP family, substitution of Trp at position 20 in maize MBP1 drastically decreased activity against *E. coli* [100]. In the trypsin inhibitor VhTI, the basic residue Arg15 was found to be critical for trypsin inhibition [90]. In the hairpinin FtAMP with a dual function (trypsin inhibition and antifungal), the substitution of the basic Arg21 for Ala or Phe abolished trypsin inhibitory activity but produced activity against elastase and α-chymotrypsin, respectively [94]. Remarkably, the antifungal activity of the peptide remained unaffected. These results clearly demonstrate that different residues (or regions of the molecule containing these residues) are responsible for different functions (enzyme inhibition, suppression of fungal growth, etc.), and that by changing particular residues, the properties of the peptide can be modified. Another spectacular example is the production of a modified hairpinin on the basis of the wheat peptide Tk-AMP-X2, which acquired the ability to block potassium channels [97]. Accordingly, identification of the residues crucial for a particular function opens up the possibility of creating molecules with novel functions.

An important issue in structure-function studies aimed at the creation of novel drugs on the basis of natural AMP sequences is the identification of minimal structural elements retaining the activity of the entire peptide. This is especially important for long peptides, since reduction of the peptide’s length is preferable to lower production costs. Considerable progress has been made in this field for defensins, in which the short γ-core motif was shown to possess antimicrobial properties. In this review, we showed that for thionins, a 45% truncated peptide of Pp-TH composed of two antiparallel α-helices of the parent peptide and stabilized by two disulfide bonds preserved the antimicrobial activity of the original thionin [36]. Thus, it is the core region responsible for the antimicrobial activity of thionin [36]. For the hevein-like peptides, the C-terminal region of the molecule was shown to be the determinant of antifungal activity against *C. cucumerinum* [78]. The truncation of the N- and C-terminal residues producing the peptide from residue 5 to residue 31 of VhTI did not affect the ability to inhibit trypsin [90]. However, truncation of “tails” in the hairpinin Sm-AMP-X resulted in a significant reduction of antifungal activity [99].

Another important concern to be addressed in the design of novel peptide drugs is to clarify the role of cysteine residues in activity and, if possible, to replace them with some other amino acid residues, given that cysteinyl residues are unstable and prone to oxidation. The data presented demonstrates that for different AMP families, the role of disulfides in peptides’ structure and activity is different. For thionins, intramolecular disulfide bonds are vital for lipid binding and toxicity [48,49,50]. Disulfides are also essential for the antimicrobial activity of α-hairpinins. In the α-hairpinin MBP-1, the replacement of cysteines with Ala dramatically decreased activity against *E. coli* [100]. In the Sm-AMP-X2 variant with only the inner disulfide bond preserved, the antifungal activity was lower than in the Sm-AMP-X1 variant with two disulfides [99]. Disulfide bonds are also important for the antifungal activity of Ib-AMP1 against most fungi [106,111]. Conversely, the deletion of cysteines had no adverse effect on the antibacterial activity of Ib-AMPs [107].

To summarize, peptides control all aspects of cellular functions in living beings. AMPs, as an integral part of the peptidome and natural antibiotics, are gaining more and more attention as emerging broad-spectrum next-generation antimicrobials. They offer much more structural and functional diversity than any other molecules, which opens up unlimited therapeutic possibilities. Synthetic short peptides corresponding to portions of the AMP molecule that retain the activity of the parent molecule and thus represent their active cores, and modified AMPs with improved properties, better therapeutic efficacy, and cheaper large-scale production are especially attractive as drug candidates. A better understanding of the modes of action of various AMPs through the finding of these minimal active structures and the identification of residues crucial for activity will inevitably culminate in the design of more effective nature-inspired peptide sequences of therapeutic value that will broaden the arsenal of anti-infective agents.

## Figures and Tables

**Table 1:** Thionins and derived peptides: sequence, net charge at pH 7.0, and biological activity. Cysteine residues are shown in red, substituted amino acids are highlighted in cyan, and modified amino acids are highlighted in green. B—α-aminobutyric acid, c—D-cysteine, and X—homocysteine. Gaps (−) were introduced to improve the alignment. Note: nd—not determined.

Peptide	Amino Acid Sequence	Net Charge at pH 7.0
	Antimicrobial Activity and Toxicity	Reference
α-Purothionin	**KS** ** CC ** **RSTLGRN** ** C ** **YNL** ** C ** **RARG--AQKL** ** C ** **AGV** ** C ** **R** ** C ** **KISSGLS** ** C ** **PKGFPK**	+10
	MIC (minimal inhibition concentration) = 3 μg/mL and MBC (minimal bactericidal concentration) = 6 μg/mL against *Pseudomonas solanacearum*; MIC = 6 μg/mL and MBC = 12 μg/mL against *Xanthomonas phaseoli*.	[33]
β-Purothionin	**KS** ** CC ** **KSTLGRN** ** C ** **YNL** ** C ** **RARG--AQKL** ** C ** **ANV** ** C ** **R** ** C ** **KLTSGLS** ** C ** **PKDFPK**	+9
	Complete growth suppression of yeasts at a concentration of 4.7 μg/mL. Lethal to mice within 40 min when administered at 0.1 mg.	[32]
	MIC = 1.5 μg/mL and MBC = 1.5 μg/mL against *P. solanacearum*; MIC = 12 μg/mL and MBC = 25 μg/mL against *X. phaseoli*.	[33]
Acetyl derivative of β-purothionin	** K ** **S** ** CC ** ** K ** **STLGRN** ** C ** **YNL** ** C ** **RARG--AQKL** ** C ** **ANV** ** C ** **R** ** C ** ** K ** **LTSGLS** ** C ** **PKDFPK**	nd
	No toxity towards yeast cells at a concentration of 11.0 μg/mL.	[32]
Succinyl derivative of β-purothionin	** K ** **S** ** CC ** ** K ** **STLGRN** ** C ** **YNL** ** C ** **RARG--AQKL** ** C ** **ANV** ** C ** **R** ** C ** ** K ** **LTSGLS** ** C ** **PKDFPK**	nd
	No toxity towards yeast cells at a concentration of 10.0 μg/mL.	[32]
Iodinated derivative of β-purothionin	**KS** ** CC ** **KSTLGRN** ** C ** ** Y ** **NL** ** C ** **RARG--AQKL** ** C ** **ANV** ** C ** **R** ** C ** **KLTSGLS** ** C ** **PKDFPK**	nd
	No toxity towards yeast cells at a concentration of 10.0 μg/mL. Scarcely lethal to mice when administered at 10.0 mg.	[32]
Nitrated derivative of β-purothionin	**KS** ** CC ** **KSTLGRN** ** C ** ** Y ** **NL** ** C ** **RARG--AQKL** ** C ** **ANV** ** C ** **R** ** C ** **KLTSGLS** ** C ** **PKDFPK**	nd
	Weakly toxic toward yeast cells at a concentration of 12.6 μg/mL. Toward mice, the efficiency is less than 30% compared to that of native purothinin.	[32]
Pp-TH	**KS** ** CC ** **RNTWARN** ** C ** **YNV** ** C ** **RLPGTISREI** ** C ** **AKK** ** C ** **D** ** C ** **KIISGTT** ** C ** **PSDYPK**	+6
	For natural Pp-TH:	
	50% hemolysis at 20 μg/mL. IC_50_ (concentration required for 50% growth inhibition) = 5.0 μg/mL against mouse B16 melanoma cells; IC_50_ = 14 μg/mL against HeLa cells; 100% lethal to mice within 4.5 min when administered at 100 μg.	[34]
	EC_50_ (Effective concentration for 50% inhibition) > 20 μM (>20 μM) against *Rhizobium meliloti*; EC_50_ = 3.1 μM (3.67 μM) against *Xanthamonas campestris* pv. *campestris*; EC_50_ = 1.7 μM against *X. campestris* pv. *translucens*; EC_50_ = 0.48 (0.23 μM) μM against *Clavibacter michiganensis* subsp. *sepedonicus* C5; EC_50_ = 0.43 μM (0.38 μM) against *Fusarium oxysporum* f. sp. *conglutinans*; EC_50_ = 0.7 μM against *Plectosphaerella cucumerina*; EC_50_ = 2.2 μM against *Botrytis cinerea*;	[35], the figures in parentheses [36]
	For synthetic Pp-TH: EC_50_ > 20 μM against *R. meliloti*; EC_50_ = 2.9 μM against *X. campestris* pv. *campestris*; EC_50_ = 1.4 μM against *X. campestris* pv. *translucens*; EC_50_ = 0.48 μM against *C. michiganensis*; EC_50_ = 0.33 μM against *F. oxysporum* f. sp. *conglutinans*; EC_50_ = 0.8 μM against *P. cucumerina*;	[35]
	EC_50_ > 20 μM against *R. meliloti*; EC_50_ = 3.65 μM against *X. campestris*; EC_50_ = 0.30 μM against *C. michiganensis*; EC_50_ = 0.36 μM against *P. cucumerina*; EC_50_ = 0.32 μM against *B. cinerea*.	[37]
Iodinated derivative of Pp-TH	**KS** ** CC ** **RNTWARN** ** C ** ** Y ** **NV** ** C ** **RLPGTISREI** ** C ** **AKK** ** C ** **D** ** C ** **KIISGTT** ** C ** **PSDYPK**	nd
	0% hemolysis at 20 μg/mL. No activity against mouse B16 melanoma and HeLa cells at 100 μg/mL; Non-lethal to mice when administered at 100 μg.	[34]
Pp-TH(D32R) or PpTHR or TH32R	**KS** ** CC ** **RNTWARN** ** C ** **YNV** ** C ** **RLPGTISREI** ** C ** **AKK** ** C ** ** R ** ** C ** **KIISGTT** ** C ** **PSDYPK**	+8
	EC_50_ = 3.3 μM (3.8 μM) against *R. meliloti*; EC_50_ = 1.2 μM (0.38 μM) against *X. campestris* pv. *campestris*; EC_50_ = 0.8 μM against *X. campestris* pv. *translucens*; EC_50_ = 0.38 μM (0.23 μM) against *C. michiganensis* subsp. *sepedonicus* C5; EC_50_ = 0.73 μM (1.52 μM) against *F. oxysporum* f. sp. *conglutinans*; EC_50_ = 1.25 μM against *P. cucumerina*; EC_50_ = 2.5 μM against *B. cinerea*;	[35], the figures in parentheses [36]
	EC_50_ = 0.8 μM against *R. meliloti*; EC_50_ = 0.3 μM against *X. campestris*; EC_50_ = 0.37 μM against *C. michiganensis*; EC_50_ = 0.36 μM against *P. cucumerina*; EC_50_ = 0.80 μM against *B. cinerea*.	[37]
PpTH(3-41)	** -- ** ** CC ** **RNTWARN** ** C ** **YNV** ** C ** **RLPGTISREI** ** C ** **AKK** ** C ** **D** ** C ** **KIISGTT** ** C **	+5
	EC_50_ > 20 μM against *R. meliloti*; EC_50_ = 7.5 μM against *X. campestris* pv. *campestris*; EC_50_ = 0.18 μM against *C. michiganensis* subsp. *sepedonicus* C5; EC_50_ = 1.73 μM against *F. oxysporum* f. sp. *conglutinans*.	[36]
PpTHR(3-41)	** -- ** ** CC ** **RNTWARN** ** C ** **YNV** ** C ** **RLPGTISREI** ** C ** **AKK** ** C ** ** R ** ** C ** **KIISGTT** ** C **	+7
	EC_50_ = 5.3 μM against *R. meliloti*; EC_50_ = 3.27 μM against *X. campestris* pv. *campestris*; EC_50_ = 0.21 μM against *C. michiganensis* subsp. *sepedonicus* C5; EC_50_ = 2.5 μM against *F. oxysporum* f. sp. *conglutinans*.	[36]
PpTH(7-32)	** ------ ** **TWARN** ** C ** **YNV** ** C ** **RLPGTISREI** ** C ** **AKK** ** C ** **D**	+3
	EC_50_ > 20 μM against *R. meliloti*; EC_50_ > 20 μM against *X. campestris* pv. *campestris*; EC_50_ = 0.63 μM against *C. michiganensis* subsp. *sepedonicus* C5; EC_50_ = 1.8 μM against *F. oxysporum* f. sp. *conglutinans*.	[36]
PpTH(7-32)b (disulfide bridges C1-C2 and C3-C4)	** ------ ** **TWARN** ** C ** **YNV** ** C ** **RLPGTISREI** ** C ** **AKK** ** C ** **D**	+3
	EC_50_ > 20 μM against *R. meliloti*; EC_50_ > 20 μM against *X. campestris* pv. *campestris*; EC_50_ = 1.8 μM against *C. michiganensis* subsp. *sepedonicus* C5; EC_50_ > 20 μM against *F. oxysporum* f. sp. *conglutinans*.	[36]
PpTHR(7-32) or TH(7-32R)	** ------ ** **TWARN** ** C ** **YNV** ** C ** **RLPGTISREI** ** C ** **AKK** ** C ** ** R **	+5
	EC_50_ = 4 μM against *R. meliloti*; EC_50_ = 7.2 μM against *X. campestris* pv. *campestris*; EC_50_ = 1.58 μM against *C. michiganensis* subsp. *sepedonicus* C5; EC_50_ = 0.5 μM against *F. oxysporum* f. sp. *conglutinans*;	[36]
	EC_50_ = 4 μM against *R. meliloti*; EC_50_ = 4.6 μM against *X. campestris*; EC_50_ = 0.80 μM against *C. michiganensis*; EC_50_ = 7.5 μM against *P. cucumerina*; EC_50_ = 0.80 μM against *B. cinerea*.	[37]
PpTH(24-32)	** ----------------------- ** **REI** ** C ** **AKK** ** C ** **D**	+1
	EC_50_ > 20 μM against *R. meliloti*; EC_50_ > 20 μM against *X. campestris* pv. *campestris*; EC_50_ > 20 μM against *C. michiganensis* subsp. *sepedonicus* C5; EC_50_ > 20 μM against *F. oxysporum* f. sp. *conglutinans*.	[36]
PpTHR(24-32) or TH(24-32R)	** ----------------------- ** **REI** ** C ** **AKK** ** C ** ** R **	+3
	EC_50_ > 20 μM against *R. meliloti*; EC_50_ > 20 μM against *X. campestris* pv. *campestris*; EC_50_ = 16 μM against *C. michiganensis* subsp. *sepedonicus* C5; EC_50_ > 20 μM against *F. oxysporum* f. sp. *conglutinans*;	[36]
	EC_50_ > 50 μM against *R. meliloti*; EC_50_ > 50 μM against *X. campestris*; EC_50_ = 21 μM against *C. michiganensis*; EC_50_ = 20 μM against *P. cucumerina*; EC_50_ = 3.50 μM against *B. cinerea*.	[37]
TH(24-32R)Abu	** ----------------------- ** **REIBAKKBR**	nd
	EC_50_ > 50 μM against *R. meliloti*; EC_50_ > 50 μM against *X. campestris*; EC_50_ = 5.13 μM against *C. michiganensis*; EC_50_ = 15.67 μM against *P. cucumerina*; EC_50_ = 4.10 μM against *B. cinerea*.	[37]
TH(24-32R)cC	** ----------------------- ** **REIcAKK** ** C ** ** R **	nd
	EC_50_ > 50 μM against *R. meliloti*; EC_50_ > 50 μM against *X. campestris*; EC_50_ = 0.90 μM against *C. michiganensis*; EC_50_ = 0.80 μM against *P. cucumerina*; EC_50_ = 0.55 μM against *B. cinerea*.	[37]
TH(24-32R)cH	** ----------------------- ** **REIcAKKXR**	nd
	EC_50_ > 50 μM against *R. meliloti*; EC_50_ > 50 μM against *X. campestris*; EC_50_ = 0.90 μM against *C. michiganensis*; EC_50_ = 0.78 μM against *P. cucumerina*; EC_50_ = 0.40 μM against *B. cinerea*.	[37]
PpTH(7-19)	** ------ ** **TWARN** ** C ** **YNV** ** C ** **RLP**	+2
	EC_50_ > 20 μM against *R. meliloti*; EC_50_ > 20 μM against *X. campestris* pv. *campestris*; EC_50_ = 15 μM against *C. michiganensis* subsp. *sepedonicus* C5; EC_50_ > 20 μM against *F. oxysporum* f. sp. *conglutinans*;	[36]
	EC_50_ > 50 μM against *R. meliloti*; EC_50_ > 50 μM against *X. campestris*; EC_50_ > 50 μM against *C. michiganensis*; EC_50_ = 29 μM against *P. cucumerina*; EC_50_ = 5.50 μM against *B. cinerea*.	[37]
PpTH(7-19)Abu	** ------ ** **TWARNBYNVBRLP**	nd
	EC_50_ > 50 μM against *R. meliloti*; EC_50_ > 50 μM against *X. campestris*; EC_50_ = 3.27 μM against *C. michiganensis*; EC_50_ = 3.97 μM against *P. cucumerina*; EC_50_ = 1.60 μM against *B. cinerea*.	[37]
PpTH(7-19)cC	** ------ ** **TWARNcYNV** ** C ** **RLP**	nd
	EC_50_ > 50 μM against *R. meliloti*; EC_50_ = 29.33 μM against *X. campestris*; EC_50_ = 0.51 μM against *C. michiganensis*; EC_50_ = 2.77 μM against *P. cucumerina*; EC_50_ = 0.20 μM against *B. cinerea*.	[37]
PpTH(7-19)cH	** ------ ** **TWARNcYNVXRLP**	nd
	EC_50_ > 50 μM against *R. meliloti*; EC_50_ = 18.33 μM against *X. campestris*; EC_50_ = 0.74 μM against *C. michiganensis*; EC_50_ = 3.50 μM against *P. cucumerina*; EC_50_ = 0.19 μM against *B. cinerea*.	[37]
PpTH(15−28)	** -------------- ** **V** ** C ** **RLPGTISREI** ** C ** **A**	+1
	EC_50_ > 20 μM against *R. meliloti*; EC_50_ > 20 μM against *X. campestris* pv. *campestris*; EC_50_ > 20 μM against *C. michiganensis* subsp. *sepedonicus* C5; EC_50_ > 20 μM against *F. oxysporum* f. sp. *conglutinans*.	[36]
TH(7-19)(24-32R)	** ------ ** **TWARN** ** C ** **YNV** ** C ** **RLP** ** - ** **+** ** -** **REI** ** C ** **AKK** ** C ** **R**	+2 and +3
	EC_50_ = 2.07 μM against *R. meliloti*; EC_50_ = 1.03 μM against *X. campestris*; EC_50_ = 0.03 μM against *C. michiganensis*; EC_50_ = 0.16 μM against *P. cucumerina*; EC_50_ = 0.08 μM against *B. cinerea*.	[37]
Viscotoxin A3	**KS** ** CC ** **PNTTGRNIYNA** ** C ** **RLTGA-PRPT** ** C ** **AKLSG** ** C ** **KIISGST** ** C ** **PSDYPK**	+6
	ED_50_ (concentration of substance inhibiting ^3^H-thymidine incorporation 50%) = 0.31 μg/mL against Yoshida sarcoma cells;	[38]
	Can penetrate into the model monolayer membrane (critical surface pressure π_c_ ≤ 39.6 μN/m).	[39]
Viscotoxin A2	**KS** ** CC ** **PNTTGRNIYNT** ** C ** **RFGGG-SREV** ** C ** **ASLSG** ** C ** **KIISAST** ** C ** **PSYPDK**	+4
	ED_50_ = 1.06 μg/mL against Yoshida sarcoma cells;	[38]
	Can penetrate into the model monolayer membrane (critical surface pressure π_c_ ≤ 32.3 μN/m).	[39]
Viscotoxin A1	**KS** ** CC ** **PSTTGRNIYNT** ** C ** **RLTGS-SRET** ** C ** **AKLSG** ** C ** **KIISAST** ** C ** **PSNYPK**	+6
	ED_50_ = 0.87 μg/mL against Yoshida cells.	[38]
Viscotoxin B	**KS** ** CC ** **PNTTGRNIYNT** ** C ** **RLGGG-SRER** ** C ** **ASLSG** ** C ** **KIISAST** ** C ** **PSDYPK**	+5
	ED_50_ = 4.58 μg/mL against Yoshida sarcoma cells;	[38]
	Penetration into the model monolayer membrane is unlikely (critical surface pressure π_c_ ≤ 27.0 μN/m).	[39]

**Table 2:** Hevein, hevein-like peptides, and derived peptides: sequence, net charge at pH 7.0, and biological activity. Cysteine residues are shown in red, and substituted amino acids are highlighted in cyan. Amino acids involved in chitin-binding are shown in green. Gaps (−) were introduced to improve the alignment.

Peptide	Amino Acid Sequence	Net Charge at pH 7.0
	Antimicrobial Activity and Toxicity	Reference
Hevein	**----EQ** ** C ** **GRQAGGKL** ** C ** **PNNL** ** CC ** ** S ** **Q** ** W ** **G** ** W ** ** C ** **GSTDE** **Y** ** C ** **SPDHN** ** C ** **QSN** ** C ** **KD---**	−2
	IC_50_ = 500 μg/mL against *B. cinerea* (MUCL 30158); IC_50_ = 600 μg/mL against *F. culmorum* (IMI 180420); IC_50_ = 1250 μg/mL against *F. oxysporum* (IMI 236441); IC_50_ = 300 μg/mL against *Phycomyces blakesleeanus* strain K1 (ATCC 5633); IC_50_ = 350 μg/mL against *Pyrenophora tritici-repentis* strain 45101; IC_50_ = 500 μg/mL against *Pyricularia oryzae* (MUCL 30166); IC_50_ = 500 μg/mL against *Septoria nodorum* (MUCL 30111); IC_50_ = 90 μg/mL against *Trichoderma hamatum* strain 10401.	[73]
LAMP-1a	**---AQK** ** C ** **GEQGRGAK** ** C ** **PN** ** C ** **L** ** CC ** **GRYGF** ** C ** **GSTPDY** ** C ** **GV-G-** ** C ** **QSQ** ** C ** **R-G** ** C ** **-**	+3
	IC_50_ = 2.7 μM (24 h after inoculation) and 5.6 μM (48 h after inoculation) against *Bipolaris sorokiniana* strain 6/10; IC_50_ = 4.1 μM (24 h after inoculation) and 6.0 μM (48 h after inoculation) against *F. oxysporum* strain 16/10.	[74]
WAMP-1a	**---AQR** ** C ** **G** **D** **QA** **R** **G** **AK** ** C ** **PN** ** C ** **L** ** CC ** **GKYGF** ** C ** **GSGDAY** ** C ** **GA-GS** ** C ** **QSQ** ** C ** **R-G** ** C ** **-**	+3
	IC_50_ = 5 μg/mL against *B. sorokiniana* strain 6/10; IC_50_ = 20 μg/mL against *B. cinerea* VKM F-85; IC_50_ = 5 μg/mL against *F. oxysporum* TSA-4; IC_50_ = 5 μg/mL against *Fusarium solani* VKM F-142; IC_50_ = 30 μg/mL against *Fusarium verticillioides* VKM F-670; IC_50_ = 10 μg/mL against *Neurospora crassa* VKM F-184; Growth inhibition of *Phytophtora infestans* strains Pril 2 and OSV 12 at 5 μM; Growth inhibition of *Pseudomonas syringae* VKM B-1546, *Clavibacter michiganensis* subsp. *michiganensis* VKM Ac-1144 and *Erwinia carotovora* subsp. *carotovora* VKM B-1247 at 2.5 μg/50μL;	[75]
	IC_50_ = 2.1 μM (24 h after inoculation) and 6.2 μM (48 h after inoculation) against *B. sorokiniana* strain 6/10; IC_50_ = 2.9 μM (24 h after inoculation) and 5.9 μM (48 h after inoculation) against *F. oxysporum* strain 16/10.	[74]
WAMP-1b	**---AQR** ** C ** **G** **D** **QA** **R** **G** **AK** ** C ** **PN** ** C ** **L** ** CC ** **GKYGF** ** C ** **GSGDAY** ** C ** **GA-GS** ** C ** **QSQ** ** C ** **R-G** ** C ** ** R **	+4
	IC_50_ = 4.9 μM against *B. sorokiniana*; IC_50_ = 16.0 μM against *F. oxysporum* F-137; IC_50_ = 18.0 μM against *Alternaria alternata*; No inhibition of *Cladosporium cucumerinum* C5 at 150 μg/mL;	[76]
	IC_50_ = 2.7 μM against *F. verticillioides* VKM F-670.	[77]
WAMP-2	**---AQR** ** C ** **G** **D** **Q** **AR** **G** **AK** ** C ** **P** **N** ** C ** **L** ** CC ** **G** **KY** **G** **F** ** C ** **GS** **GDA** **Y** ** C ** **G** ** K ** **-GS** ** C ** **QSQ** ** C ** **R-G** ** C ** **R**	+5
	IC_50_ = 6.6 μM against *B. sorokiniana* KrD-81*;* IC_50_ = 8.8 μM against *F. oxysporum* F37; IC_50_ = 52.9 μM against *F. culmorum* OR-02-37; IC_50_ = 23.0 μM against *A. alternata* MRD-12; IC_50_ = 8.0 μM against *C. cucumerinum* C5;	[78]
	IC_50_ = 2.2 μM against *F. verticillioides* VKM F-670.	[77]
WAMP-3.1	**---AQR** ** C ** **G** **D** **Q** **AR** **G** **AK** ** C ** **P** **N** ** C ** **L** ** CC ** **G** **KY** **G** **F** ** C ** **GS** **GDA** **Y** ** C ** **G** ** E ** **-GS** ** C ** **QSQ** ** C ** **R-G** ** C ** **R**	+3
	IC_50_ = 4.8 μM against *B. sorokiniana* KrD-81*;* IC_50_ = 6.8 μM against *F. oxysporum* F37; IC_50_ = 17.8 μM against *A. alternata* MRD-12; No inhibition of *C. cucumerinum* C5 at 150 μg/mL;	[78]
	IC_50_ = 3.5 μM against *F. verticillioides* VKM F-670.	[77]
WAMP-4	**---AQR** ** C ** **G** **D** **Q** **AR** **G** **AK** ** C ** **P** **N** ** C ** **L** ** CC ** **G** **KY** **G** **F** ** C ** **GS** **GDA** **Y** ** C ** **G** ** N ** **-GS** ** C ** **QSQ** ** C ** **R-G** ** C ** **R**	+4
	IC_50_ = 5.2 μM against *B. sorokiniana* KrD-81; IC_50_ = 11.2 μM against *F. oxysporum* F37; IC_50_ = 23.2 μM against *A. alternata* MRD-12; IC_50_ = 12.5 μM against *C. cucumerinum* C5;	[78]
	IC_50_ = 3.0 μM against *F. verticillioides* VKM F-670.	[77]
WAMP-5	**---AQR** ** C ** **G** **D** **Q** **AR** **G** **AK** ** C ** **P** **N** ** C ** **L** ** CC ** **G** **KY** **G** **F** ** C ** **GS** **GDA** **Y** ** C ** **G** ** V ** **-GS** ** C ** **QSQ** ** C ** **R-G** ** C ** **R**	+4
	IC_50_ = 5.4 μM against *B. sorokiniana*; IC_50_ = 12.1 μM against *F. oxysporum* F-137; IC_50_ = 13.8 μM against *A. alternata*; IC_50_ = 11.6 μM against *C. cucumerinum*.	[76]
WAMP-N	** --- ** **AQR** ** C ** **G** **D** **Q** **AR** **G** **AK** ** C **	+2
	IC_50_ = 53.5 μM against *B. sorokiniana* KrD-81; IC_50_ = 174.6 μM against *F. oxysporum* F37; IC50 = 243.5 μM against *F. culmorum* OR-02-37; IC_50_ > 500 μM against *Fusarium avenaceum* Br-04-60; IC_50_ = 75.3 μM against *A. alternata* MRD-12; IC_50_ = 205.5 μM against *C. cucumerinum* C5; IC_50_ = 161.5 μM against *Parastagonospora nodorum* B-9/47.	[78]
WAMP-G1	** ------------------- ** **L** ** CC ** **G** **KY** **G** **F** ** C ** **GS** **G**	+1
	IC_50_ = 228.7 μM against *B. sorokiniana* KrD-81; IC_50_ > 500 μM against *F. oxysporum* F37; IC_50_ > 500 μM against *F. culmorum* OR-02-37; No activity against *F. avenaceum* Br-04-60 at 400 μg/mL; IC_50_ > 500 μM against *A. alternata* MRD-12; IC_50_ > 500 μM against *C. cucumerinum* C5; IC_50_ > 500 μM against *P. nodorum* B-9/47.	[78]
WAMP-G2	** -------------------- ** ** CC ** **G** **KY** **G** **F** ** C ** **GS** **GDA** **Y** ** C **	0
	IC_50_ = 127.3 μM against *B. sorokiniana* KrD-81; IC_50_ = 255.1 μM against *F. oxysporum* F37; IC_50_ > 500 μM against *F. culmorum* OR-02-37; IC_50_ = 393.1 μM against *F. avenaceum* Br-04-60; IC_50_ = 94.9 μM against *A. alternata* MRD-12; IC_50_ = 267.4 μM against *C. cucumerinum* C5; IC_50_ = 276.5 μM against *P. nodorum* B-9/47.	[78]
WAMP-C	** ----------------------------------- ** **G** **K-GS** ** C ** **QSQ** ** C ** **R-G** ** C ** **R**	+3
	IC_50_ = 313.6 μM against *B. sorokiniana* KrD-81; IC_50_ > 500 μM against *F. oxysporum* F37; IC_50_ > 500 μM against *F. culmorum* OR-02-37; No activity against *F. avenaceum* Br-04-60 at 400 μg/mL; IC_50_ = 401.9 μM against *A. alternata* MRD-12; IC_50_ = 3.9 μM against *C. cucumerinum* C5; IC_50_ = 240.7 μM against *P. nodorum* B-9/47.	[78]
Ee-CBP	**----QQ** ** C ** **GRQAGNRR** ** C ** **ANNL** ** CC ** **SQYGY** ** C ** **GRTNEY** ** CC ** **TSQG** ** C ** **QSQ** ** C ** **RR-** ** C ** **G**	+5
	IC_50_ = 3 μg/mL (0.6 μM) against *Alternaria brassicicola* MUCL 20297; IC_50_ = 1 μg/mL (0.2 μM) against *B. cinerea* MUCL 6492; IC_5_0 = 3 μg/mL (0.6 μM) against *F. culmorum* IMI 180420; IC_50_ = 15 μg/mL (3 μM) against *F. oxysporum* f. sp. *cubense*; IC_50_ = 5 μg/mL (1 μM) against *F. oxysporum* f. sp. *matthiolae* CBS 247.61; IC_50_ = 6 μg/mL (1.2 μM) against *Mycosphaerella eumusae*; IC_50_ = 2 μg/mL (0.4 μM) against *N. crassa* FGSC 2489; IC_50_ = 33 μg/mL (6.6 μM) against *Phoma exigua* CBS 431.74; IC_50_ = 25 μg/mL (5 μM) against *Phytophthora cryptogea* CBS 418.71; IC_50_ = 33 μg/mL (6.6 μM) against *Pythium ultimum* MUCL 30159; IC_50_ = 25 μg/mL (5 μM) against *Rhizoctonia solani* CBS 207.84; IC_50_ = 100 μg/mL (20 μM) against *Trichoderma hamatum* ATCC 20765; IC_50_ = 2 μg/mL against *Bacillus megaterium* ATCC 13632; IC_50_ = 7 μg/mL against *Sarcina lutea* ATCC 9341.	[79], the figures in parentheses [62]
EAFP1	**----QT** ** C ** **ASR** ** C ** **P-RP** ** C ** **NAGL** ** CC ** **SIYGY** ** C ** **GSGNAY** ** C ** **GA-GN** ** C ** **R** ** C ** **Q** ** C ** **RG---**	+4
	IC_50_ = 155 μg/mL against *Aculops lycopersici*; IC_50_ = 56 μg/mL against *Fusarium moniliforme*; IC_50_ = 46 μg/mL against *F. oxysporum*; IC_50_ = 35 μg/mL against *Colletotrichum gossypii*; No effect on the growth of *Bacillus megaterium* and *Pseudomonas syringae*.	[80]
EAFP2	**----QT** ** C ** **ASR** ** C ** **P-RP** ** C ** **NAGL** ** CC ** **SIYGY** ** C ** **GSGAAY** ** C ** **GA-GN** ** C ** **R** ** C ** **Q** ** C ** **RG---**	+4
	IC_50_ = 109 μg/mL against *A. lycopersici*; IC_50_ = 18 μg/mL against *F. moniliforme*; IC_50_ = 94 μg/mL against *F. oxysporum*; IC_50_ = 56 μg/mL against *C. gossypii*; No effect on the growth of *B. megaterium* and *P. syringae*.	[80]
Pn-AMP1	**----QQ** ** C ** **GRQASGRL** ** C ** **GNRL** ** CC ** **SQWGY** ** C ** **GSTASY** ** C ** **G--AG** ** C ** **QSQ** ** C ** **RS---**	+4
	IC_50_ = 16 μg/mL against *B. cinerea*; IC_50_ = 10 μg/mL against *Colletotrichum langenarium*; IC_50_ = 11 μg/mL against *Sclerotinia sclerotiorum*; IC_50_ = 10 μg/mL against *F. oxysporum*; IC_50_ = 26 μg/mL against *R. solani*; IC_50_ = 5 μg/mL against *Phytophthora capsici*; IC_50_ = 3 μg/mL against *Phytophthora parasitica* cv. *nicotianae*; IC_50_ = 14 μg/mL against *Saccharomyces cerevisiae* EGY48; No activity against *Escherichia coli*, *Agrobacterium tumefaciens* and cultured cells *Spodoptera frugiperda* 9 and MA104 at concentrations up to 200 μg/mL; IC_50_ = 38 μg/mL against *Bacillus subtilis*.	[81]
Pn-AMP2	**----QQ** ** C ** **GRQASGRL** ** C ** **GNRL** ** CC ** **SQWGY** ** C ** **GSTASY** ** C ** **G--AG** ** C ** **QSQ** ** C ** **R----**	+4
	IC_50_ = 2 μg/mL against *B. cinerea*; IC_50_ = 4 μg/mL against *C. langenarium*; IC_50_ = 3 μg/mL against *S. sclerotiorum*; IC_50_ = 2.5 μg/mL against *F. oxysporum*; IC_50_ = 75 μg/mL against *R. solani*; IC_50_ = 0.6 μg/mL against *P. capsici*; IC_50_ = 2 μg/mL against *P. parasitica* cv. *nicotianae*; IC_50_ = 8 μg/mL against *S. cerevisiae* EGY48; IC_50_ = 2.5 μg/mL against *Pythium* spp.; No activity against Gram-negative *E. coli*, *A. tumefaciens* and cultured cells *S. frugiperda* 9 and MA104 at concentrations up to 200 μg/mL; IC_50_ = 20 μg/mL against *B. subtilis*.	[81]
Fa-AMP1	**----AQ** ** C ** **GAQGGGAT** ** C ** **PGGL** ** CC ** **SQWGW** ** C ** **GSTPKY** ** C ** **G--AG** ** C ** **QSN** ** C ** **K----**	+2
	IC_50_ = 19 μg/mL against *F. oxysporum* IFO 6384; IC_50_ = 36 μg/mL against *Geotrichum candidum*; IC_50_ = 11 μg/mL against *Erwinia carotovora* subsp. *carotovora* MAFF 106567; IC_50_ = 24 μg/mL against *Agrobacterium radiobacter* MAFF 520028*;* IC_50_ = 20 μg/mL against *Agrobacterium rhizogenes* MAFF 210265; IC_50_ = 14 μg/mL against *C. michiganensis* subsp. *michiganensis* MAFF 301044; IC_50_ = 13 μg/mL against *Curtobacterium flaccumfaciens* pv. oorti MAFF 301203.	[82]
Fa-AMP2	**----AQ** ** C ** **GAQGGGAT** ** C ** **PGGL** ** CC ** **SQWGW** ** C ** **GSTPKY** ** C ** **G--AG** ** C ** **QSN** ** C ** **R----**	+2
	IC_50_ = 29 μg/mL against *F. oxysporum* IFO 6384; IC_50_ = 25 μg/mL against *G. candidum*; IC_50_ = 15 μg/mL against *E. carotovora* subsp. *carotovora* MAFF 106567; IC_50_ = 17 μg/mL against *A. radiobacter* MAFF 520028*;* IC_50_ = 24 μg/mL against *A. rhizogenes* MAFF 210265; IC_50_ = 17 μg/mL against *C. michiganensis* subsp. *michiganensis* MAFF 301044; IC_50_ = 15 μg/mL against *C. flaccumfaciens* pv. oorti MAFF 301203.	[82]
mO1	**----QN** ** C ** **GRQAGNRA** ** C ** **ANQL** ** CC ** **SQYGF** ** C ** **GSTSEY** ** C ** **SRANG** ** C ** **QSN** ** C ** **RGG--**	+3
	IC_50_ = 25.51 μg/mL against *A. alternata* CICC 2465; IC_50_ = 60.43 μg/mL against *A. brassicicola* CICC 2646; No activity against *Curvularia lunata* CICC 40301, *F. oxysporum* CICC 2532, *Aspergillus niger* CICC 2089, *Verticillium dahilae* CICC 2534, *R. solani* CICC 40259 at a concentration of 70 μg/mL; No significant cytotoxic effect on Vero cells with concentrations up to 100 μM.	[58]
vH2	**----FQ** ** C ** **GRQAGGAR** ** C ** **SNGL** ** CC ** **SQFGY** ** C ** **GSTPPY** ** C ** **GA-GQ** ** C ** **QSQ** ** C ** **-----**	+2
	IC_50_ = 21.87 μg/mL against *A. alternata* CICC 40292; IC_50_ = 16.10 μg/mL against *C. lunata* CICC 40301; IC_50_ = 5.05 μg/mL against *F. oxysporum* CICC 2532; IC_50_ = 1.77 μg/mL against* R. solani* CICC 40259.	[54]
gB5	**---DPT** ** C ** **SKL-GDFK** ** C ** **NPGR** ** CC ** **SKFNY** ** C ** **GSTAAY** ** C ** **GR-GN** ** C ** **IAQ** ** C ** **P----**	+3
	IC_50_ = 6.8 μg/mL against *A. niger*; IC_50_ = 10.0 μg/mL against *C. lunata* CICC 40301; IC_50_ = 69.2 μg/mL against* F. oxysporum* CICC 2532; IC_50_ = 20.0 μg/mL against* R. solani* CICC 40259.	[53]
Ac-AMP1	**---VGE** ** C ** **---VRG-R** ** C ** **PSGM** ** CC ** **SQFGY** ** C ** **GKGPKY** ** C ** **G--------------**	+3
	IC_50_ = 7 μg/mL against *A. brassicola*; IC_50_ = 8 μg/mL against *A. pisi*; IC_50_ = 10 μg/mL against *B. cinerea*; IC_50_ = 8 μg/mL against *C. lindemuthianum*; IC_50_ = 2 μg/mL against *F. culmorum*; IC_50_ = 7 μg/mL against *T. hamatum*; IC_50_ = 6 μg/mL against *V. dahlia*; IC_50_ = 40 μg/mL against *B. megaterium*; IC_50_ = 250 μg/mL against *S. lutea*.	[83]
Ac-AMP2	**---VGE** ** C ** **---VRG-R** ** C ** **PSGM** ** CC ** **SQ** ** F ** **G** ** Y ** ** C ** **GKGPK** ** Y ** ** C ** **GR-------------**	+4
	IC_50_ = 4 μg/mL against *A. brassicola*; IC_50_ = 8 μg/mL against *A. pisi*; IC_50_ = 8 μg/mL against *B. cinerea*; IC_50_ = 8 μg/mL against *C. lindemuthianum*; IC_50_ = 2 μg/mL against *F. culmorum*; IC_50_ = 3 μg/mL against *T. hamatum*; IC_50_ = 8 μg/mL against *V. dahliae*; IC_50_ = 10 μg/mL against *B. megaterium*; IC_50_ = 40 μg/mL against *S. lutea*;	[83]
	IC_50_ = 50 μg/mL against *A. brassicicola* MUCL 20297; IC_50_ = 2 μg/mL against *B. cinerea* MUCL 6492; IC_50_ = 6 μg/mL against *F. culmorum* IMI 180420; IC_50_ = 100 μg/mL against *F. oxysporum* f. sp. *cubense*; IC_50_ = 30 μg/mL against *F. oxysporum* f. sp. *matthiolae* CBS 247.61; IC_50_ = 8 μg/mL against *Mycosphaerella eumusae*; IC_50_ = 3 μg/mL against *N. crassa* FGSC 2489; IC_50_ = 30 μg/mL against *Phoma exigua* CBS 431.74; IC_50_ = 50 μg/mL against *Phytophthora cryptogea* CBS 418.71; IC_50_ = 95 μg/mL against *Pythium ultimum* MUCL 30159; IC_50_ = 100 μg/mL against *R. solani* CBS 207.84; IC_50_ = 100 μg/mL against *T. hamatum* ATCC 20765; IC_50_ = 7 μg/mL against *B. megaterium* ATCC 13632; IC_50_ = 20 μg/mL against *S. lutea* ATCC 9341.	[79]
Ar-AMP	**---AGE** ** C ** **---VQG-R** ** C ** **PSGM** ** CC ** **SQFGY** ** C ** **GRGPKY** ** C ** **GR-------------**	+3
	Growth inhibition of *F. culmorum* at 3.5 μM; Growth inhibition of *Helminthosporium sativum* and *B. cinerea* at 10.6 μM; Growth inhibition of *Alternaria consortiale* at 31.8 μM; No growth inhibition of *R. solani* at 286.0 μM.	[84]
IWF4	**---SGE** ** C ** **NMYG---R** ** C ** **PPGY** ** CC ** **SKFGY** ** C ** **GVGRAY** ** C ** **G--------------**	+2
	IC_50_ = 0.7 μM against *Cercospora beticola* isolate FC573 (oxidized and nonoxidized IWF4 showed the same activity level).	[55]
SmAMP3	**VGPGGE** ** C ** **GGRFGG--** ** C ** **AGGQ** ** CC ** **SRFGF** ** C ** **GSGPKY** ** C ** **AH** **-------------**	+2
	IC_50_ = 5.4 μM against *A. niger*; IC_50_ = 2.0 μM against *B. sorokiniana*; IC_50_ = 1.6 μM against *B. cinerea*; IC_50_ = 3.7 μM against *F. solani*; IC_50_ = 5.0 μM against *A. alternata*.	[85]
SmAMP1.1	**SGPNGQ** ** C ** **GPGWGG--** ** C ** **RGGL** ** CC ** **SQYGY** ** C ** **GSGPKY** ** C ** **AH-------------**	+2
	IC_50_ = 2.4 μM against *B. cinerea*; IC_50_ = 3.5 μM against *F. solani*; IC_50_ = 2.6 μM against *A. alternata*.	[85]

**Table 3:** The α-hairpinins and derived peptides: sequence, net charge at pH 7.0, and biological activity. Cysteine residues are shown in red, and substituted amino acids are highlighted in cyan. Gaps (−) were introduced to improve the alignment. Note: O—hydroxyproline; *—both figures are given in the original article.

Peptide	Amino Acid Sequence	Net Charge at pH 7.0
	Biological Activity	Reference
MBP-1	**------** **RSGRGE** ** C ** **RRQ** ** C ** **LRRHEGQPWETQE** ** C ** **MRR** ** C ** **RRRG---** **-**	+7
	99.9% growth inhibition of *E. coli* DH5 at a concentration of 3 μg/mL; 81% growth inhibition of *C. michiganense* ssp. *nebraskense* at a concentration of 30 μg/mL; Almost complete growth inhibition of *F. graminearum*, *Sclerotina sclerotiorum*, and *Alternaria longipes* at 60 μg/mL; Almost complete growth inhibition of *Sclerotina trifoliorum* at 30 μg/mL; Growth inhibition of *F. moniliforme* at 60 μg/mL; Weak growth inhibition of *Aspergillus flavus* at 60 μg/mL;	[101]
	MIC = 50 μM against *E. coli* DH5-α.	[100]
Var 1 (W20A)	**------RSGRGE** ** C ** **RRQ** ** C ** **LRRHEGQPAETQE** ** C ** **MRR** ** C ** **RRRG----**	+7
	MIC > 400 μM against *E. coli* DH5-α.	[100]
Var 2	**------RSGRGEARRQALRRHEGQPWETQEAMRRARRRG----**	+7
	MIC > 400 μM against *E. coli* DH5-α.	[100]
VhTI	**------NTDPEQ** ** C ** **KVM** ** C ** **YAQRHSSPELLRR** ** C ** **LDN** ** C ** **EKEHD---**	−2
	Trypsin inhibitor.	[90]
VhTI (5-31)	** ---------- ** **EQ** ** C ** **KVM** ** C ** **YAQRHSSPELLRR** ** C ** **LDN** ** C ** **EK**	+1
	Trypsin inhibitor.	[90]
FtAMP	**GSSEKPQQELEE** ** C ** **QNV** ** C ** **RMKRWSTEMVHR** ** C ** **EKK** ** C ** **EEKFERQQR**	+1
	K_i_ (trypsin) = 1.90 × 10^−9^ M; No inhibitory activity against elastase or α-chymotrypsin. MIC > 128 μM against *E. coli* BNCC 337271; MIC = 128 μM against *B. subtilis* BNCC 124990; MIC = 128 μM against *S. aureus* BNCC 186335; MIC = 16 μM against *F. oxysporum* BNCC 164775; MIC = 8 μM against *Rhizopus* sp. BNCC 147803; MIC = 8 μM against *Trichoderma koningii* BNCC 189731.	[94]
FtAMP-R21A	**GSSEKPQQELEE** ** C ** **QNV** ** C ** **RMKAWSTEMVHR** ** C ** **EKK** ** C ** **EEKFERQQR**	0
	17.3% tripsin-inhibitory activity of FtAMP; K_i_ (elastase) = 2.47 × 10^−9^ M; MIC > 128 μM against *E. coli* BNCC 337271; MIC = 128 μM against *B. subtilis* BNCC 124990; MIC = 128 μM against *S. aureus* BNCC 186335; MIC = 16 μM against *F. oxysporum* BNCC 164775; MIC = 8 μM against *Rhizopus* sp. BNCC 147803; MIC = 8 μM against *T. koningii* BNCC 189731.	[94]
FtAMP-R21F	**GSSEKPQQELEE** ** C ** **QNV** ** C ** **RMKFWSTEMVHR** ** C ** **EKK** ** C ** **EEKFERQQR**	0
	14.7% tripsin-inhibitory activity of FtAMP; K_i_ (α-chymotrypsin) = 2.73 × 10^−9^ M; MIC > 128 μM against *E. coli* BNCC 337271; MIC = 128 μM against *B. subtilis* BNCC 124990; MIC = 128 μM against *S. aureus* BNCC 186335; MIC = 16 μM against *F. oxysporum* BNCC 164775; MIC = 8 μM against *Rhizopus* sp. BNCC 147803; MIC = 8 μM against *T. koningii* BNCC 189731.	[94]
Sm-AMP-X	**----VDPDVRAYCKHQCMSTRGDQARKICESVCMRQD------**	+1
	IC_50_ = 12.5 μM against *A. alternata* strain DVZ; IC_50_ = 4.0 μM against *A. niger* VKM F-33; IC_50_ > 32.0 μM against *B. sorokiniana* 6/10; IC_50_ = 16.2 μM against *B. cinerea* SGR-1; IC_50_ = 5.4 μM against *F. oxysporum* 16/10; IC_50_ = 7.2 μM against *F. solani* IVK; IC_50_ > 32.0 μM against *P. infestans* OSV 12; IC_50_ > 32.0 μM against *P. ultimum* F-1506; No activity against *C. michiganensis* subsp*. michiganensis* VKM Ac-1144, *E. carotovora* subsp. *carotovora* VKM B-1247, *E. coli* XL1-blue, and *P. syringae* VKM B-1546 at concentrations up to 40 μM.	[99]
Sm-AMP-L	**----VDPDVRAYCKHQCLSTRGDQARKICESVCLRQD------**	+1
	IC_50_ = 12.9 μM against *A. alternata* strain DVZ; IC_50_ = 3.6 μM against *A. niger* VKM F-33; IC_50_ > 32.0 μM against *B. sorokiniana* 6/10; IC_50_ = 18.0 μM against *B. cinerea* SGR-1; IC_50_ = 6.1 μM against *F. oxysporum* 16/10; IC_50_ = 7.2 μM against *F. solani* IVK; IC_50_ > 32.0 μM against *P. infestans* OSV 12; IC_50_ > 32.0 μM against *P. ultimum* F-1506.	[99]
Sm-AMP-X1	** ------------ ** ** C ** **KHQ** ** C ** **MSTRGDQARKI** ** C ** **ESV** ** C ** **M**	+2
	IC_50_ > 32.0 μM against *A. alternata* strain DVZ; IC_50_ = 8.0 μM against *A. niger* VKM F-33; IC_50_ > 32.0 μM against *B. sorokiniana* 6/10; IC_50_ > 32.0 μM against *B. cinerea* SGR-1; IC_50_ = 24.4 μM against *F. oxysporum* 16/10; IC_50_ = 19.0 μM against *F. solani* IVK; IC_50_ > 32.0 μM against *P. infestans* OSV 12; IC_50_ > 32.0 μM against *P. ultimum* F-1506.	[99]
Sm-AMP-X2	** ---------------- ** ** C ** **MSTRGDQARKI** ** C ** **E**	+1
	IC_50_ > 32.0 μM against *A. alternata* strain DVZ; IC_50_ = 16.9 μM against *A. niger* VKM F-33; IC_50_ > 32.0 μM against *B. sorokiniana* 6/10; IC_50_ > 32.0 μM against *B. cinerea* SGR-1; IC_50_ = 25.0 μM against *F. oxysporum* 16/10; IC_50_ = 22.5 μM against *F. solani* IVK; IC_50_ > 32.0 μM against *P. infestans* OSV 12; IC_50_ > 32.0 μM against *P. ultimum* F-1506.	[99]
Tk-AMP-X1	**--------TDDR** ** C ** **ERM** ** C ** **QHYHDRREKKQ** ** C ** **MKG** ** C ** **RYGESD---**	+1
	IC_50_ = 7.5 μg/mL against *F. graminearum*; IC_50_ = 15.0 μg/mL against *F. verticillioides*; IC_50_ = 30 μg/mL against *Diplodia maydis*; IC_50_ > 30 μg/mL against *Colletotrichum graminicola*.	[96]
Tk-AMP-X2	**--------ADDR** ** C ** **ERM** ** C ** **QRYHDRREKKQ** ** C ** **MKG** ** C ** **RYG------**	+4
	IC_50_ = 7.5 μg/mL against *F. graminearum*; IC_50_ = 10.0 μg/mL against *F. verticillioides*; IC_50_ = 17 μg/mL against *D. maydis*; IC_50_ > 30 μg/mL against *C. graminicola*;	[96]
	No activity against the tested voltage-gated potassium channels (members of the Shaker (Kv1.1–Kv1.6 and Shaker IR), Shab (Kv2.1), Shaw (Kv3.1), and erg (hERG) families) even at concentrations up to 250 μM.	[97]
Tk-hefu	**--------ADDR** ** C ** ** Y ** **RM** ** C ** **QRYHDRREKKQ** ** C ** ** KE ** **G** ** C ** **RYG------**	+4
	Selectively targets members of the Shaker family: at 40 μM, it inhibited the potassium currents through Kv1.2, Kv1.3, and Kv1.6 channels by 8.3, 58.4, and 7.3%, respectively. No activity on other channels. It blocked Kv1.3 channels with similar potency (IC_50_ 34.0 μM) to κ-hefutoxin 1 (IC_50_~40.0 μM).	[97]
EcAMP1	**------GSGRGS** ** C ** **RSQ** ** C ** **MRRHEDEPWRVQE** ** C ** **VSQ** ** C ** **RRRRGGGD**	+4
	EC_50_ = 16.0 μM against *A. alternata*; EC_50_ = 14.0 μM against *A. solani*; EC_50_ > 32 μM against *A. niger*; EC_50_ = 18.2 μM against *B. sorokiniana*; EC_50_ > 10 μM against *C.graminicola*; EC_50_ > 10 μM against *D. maydis*; EC_50_ = 4.5 μM against *F. graminearum*; EC_50_ = 8.5 μM against *F. oxysporum*; EC_50_ = 4.0 μM against *F. solani*; EC_50_ = 8.1 μM against *F. verticillioides*; EC_50_ = 6.0 μM against *Phoma betae*; EC_50_ = 16.3 μM against *P. infestans*; EC_50_ = 12.0 μM against *Pythium debaryanum*; EC_50_ = 14.4 μM against *P. ultimum*; EC_50_ > 32 μM against *Trichoderma album*;	[91]
	IC_50_ = 3.8 μM against *F. solani*;	[102]
	IC_50_ = 5 μM against *S. aureus*; No activity against *E. coli* and *P. aeruginosa* at 80 μM; IC_50_ = 0.625 μM (MIC99 = 1.25 μM) against *Candida albicans*; IC_50_ = 6.8 (5.0)* μM against *F. graminearum VKM F-1668*; IC_50_ = 12.9 (9.4)* μM against *F. oxysporum TSKHA-4*; IC_50_ = 5.4 (5.6)* μM against *F. solani*; IC_50_ > 32 μM against *A. niger VKM F-33*; IC_50_ = 25.7 μM against *B. sorokiniana VKM F-1446*; IC_50_ = 18.4 μM against *A. alternata*.	[103]
EcAMP1-X1	** ------------ ** ** C ** **RSQ** ** C ** **MRRHEDEPWRVQE** ** C ** **VSQ** ** C **	0
	IC_50_ = 9.0 μM against *F. graminearum VKM F-1668*; IC_50_ = 15.4 μM against *F. oxysporum TSKHA-4*; IC_50_ = 6.9 μM against *F. solani*; IC_50_ > 32 μM against *A. niger VKM F-33*; IC_50_ > 32.0 μM against *B. sorokiniana VKM F-1446*; IC_50_ = 21.1 μM against *A. alternata*; No activity against *S. aureus*, *E. coli*, *P. aeruginosa,* and *C. albicans* at 80 μM.	[103]
EcAMP1-X2	** ---------------- ** ** C ** **MRRHEDEPWRVQE** ** C **	−1
	IC_50_ = 18.1 μM against *F. graminearum VKM F-1668*; IC_50_ = 23.2 μM against *F. oxysporum TSKHA-4*; IC_50_ = 11.0 μM against *F. solani*; IC_50_ > 32 μM against *A. niger VKM F-33*; IC_50_ > 32.0 μM against *B. sorokiniana VKM F-1446*; IC_50_ > 32.0 μM against *A. alternata*; No activity against *S. aureus*, *E. coli*, *P. aeruginosa,* and *C. albicans* at 80 μM.	[103]
EcAMP1-X3	**------GSGRGS** ** C ** **RSQ** ** C ** **MRRHEDEPARVQE** ** C ** **VSQ** ** C ** **RRRRGGGD**	+4
	IC_50_ = 9.9 μM against *F. graminearum VKM F-1668*; IC_50_ = 15.0 μM against *F. oxysporum TSKHA-4*; IC_50_ = 8.6 μM against *F. solani*.	[103]
EcAMP1-X4	** ------ ** **GSGRGS** ** C ** **RSQ** ** C ** **MRRHEDEPWRVQE** ** C ** **VSQ** ** C ** **RR**	+3
	IC_50_ = 8.5 μM against *F. graminearum VKM F-1668*; IC_50_ = 15.8 μM against *F. oxysporum TSKHA-4*; IC_50_ = 7.8 μM against *F. solani*.	[103]
EcAMP1-Hyp	**------GSGRGS** ** C ** **RSQ** ** C ** **MRRHEDEOWRVQE** ** C ** **VSQ** ** C ** **RRRRGGGD**	nd
	IC_50_ = 5.4 μM against *F. solani*.	[102]

**Table 4:** Ib-AMP1-4 and derived peptides: sequence, net charge at pH 7.0, and antimicrobial activity. Cysteine residues are shown in red, and substituted amino acids are highlighted in cyan. Gaps (−) were introduced to improve the alignment. Note: nd—not determined; *—both figures are given in the original article; *a*—Ala peptoid residue (Nala) (CH3–NH–CH2–COOH); *k*—Lys peptoid residue (Nlys) (NH2–CH2–CH2–CH2–CH2–NH–CH2–COOH); <E—pyroGlu; B—α-amino butyric acid; L- and D-amino acids are indicated by capital and small letters, respectively.

Peptide	Amino Acid Sequence	Net Charge at pH 7.0
	Antimicrobial Activity and Toxicity	Reference
Ib-AMP2	**QYGRR** ** CC ** **N** **W** **GPGRRY** ** C ** **KRW** ** C **	+6
	IC_50_ = 12 μg/mL against *Alternaria longipes* CBS62083; IC_50_ = 25 μg/mL against *B. cinerea* K1147; IC_50_ = 6 μg/mL against *Cladosporium sphaerospermum* K0791; IC_50_ = 6 μg/mL against *F. culmorum* K0311; IC_50_ = 6 μg/mL against *Penicillium digitatum* K0879; IC_50_ = 12 μg/mL against *Trichoderma viride* K1127; IC_50_ = 12 μg/mL against *Verticillium alboatrum* K0937; No activity on erythrocytes and cultured fibroblasts at a concentration of 200 μg/mL.	[9]
Ib-AMP3	**QYRHR** ** CC ** **AWGPGRKY** ** C ** **KRW** ** C **	+6
	IC_50_ = 6 μg/mL against *A. longipes* CBS62083; IC_50_ = 6 μg/mL against *B. cinerea* K1147; IC_50_ = 3 μg/mL against *C. sphaerospermum* K0791; IC_50_ = 6 μg/mL against *F. culmorum* K0311; IC_50_ = 3 μg/mL against *P. digitatum* K0879; IC_50_ = 12 μg/mL against *T. viride* K1127; IC_50_ = 6 μg/mL against *V. alboatrum* K0937.	[9]
Ib-AMP4	**QWGRR** ** CC ** **GWGPGRRY** ** C ** **RRW** ** C **	+6
	IC_50_ = 3 μg/mL against *A. longipes* CBS62083; IC_50_ = 6 μg/mL against *B. cinerea* K1147; IC_50_ = 1 μg/mL against *C. sphaerospermum* K0791; IC_50_ = 1 μg/mL against *F. culmorum* K0311; IC_50_ = 3 μg/mL against *P. digitatum* K0879; IC_50_ = 6 μg/mL against *T. viride* K1127; IC_50_ = 6 μg/mL against *V. alboatrum* K0937; IC_50_ = 5 μg/mL against *Bacillus subtilis* JHCC 55331; IC_50_ = 5 μg/mL against *Micrococcus luteus* ATCC 9341; IC_50_ = 20 μg/mL against *Staphylococcus aureus* ATCC 25923; IC_50_ = 5 μg/mL against *Streptococcus faecalis* ATCC 29212; IC_50_ > 500 μg/mL against *E. coli* HB101; IC_50_ > 500 μg/mL against *Proteus vulgaris* JHCC 558711; IC_50_ > 100 μg/mL against *Pseudomonas solanacearum* R48/a; IC_50_ > 100 μg/mL against *Erwinia amylovora* CFBP1430; IC_50_ = 6 μg/mL against *X. campestris* INRA 10342; IC_50_ = 15 μg/mL against *X. oryzae* ETH 698; No activity on erythrocytes and cultured fibroblasts at a concentration of 200 μg/mL.	[9]
Ib-AMP1	**QWGRR** ** CC ** **GWGPGRRY** ** C ** **VRW** ** C **	+5
	IC_50_ = 3 μg/mL against *A. longipes* CBS62083; IC_50_ = 12 μg/mL against *B. cinerea* K1147; IC_50_ = 1 μg/mL against *C. sphaerospermum* K0791; IC_50_ = 1 μg/mL against *F. culmorum* K0311; IC_50_ = 3 μg/mL against *P. digitatum* K0879; IC_50_ = 6 μg/mL against *T. viride* K1127; IC_50_ = 3 μg/mL against *V. alboatrum* K0937; IC_50_ = 10 μg/mL against *B. subtilis* JHCC 55331; IC_50_ = 10 μg/mL against *M. luteus* ATCC 9341; IC_50_ = 30 μg/mL against *S. aureus* ATCC 25923; IC_50_ = 6 μg/mL against *S. faecalis* ATCC 29212; IC_50_ > 500 μg/mL against *E. coli* HB101; IC_50_ > 500 μg/mL against *P. vulgaris* JHCC 558711; IC_50_ > 500 μg/mL against *P. solanacearum* R48/a;	[9]
	MIC = 2.5 μM against *Aspergillus flavus* KCTC 1375; MIC = 5 μM against *Candida albicans*.	[106]
Ib-AMP1 (reduced form)	**QWGRR** ** CC ** **GWGPGRRY** ** C ** **VRW** ** C **	+5
	MIC = 10 μM against *A. flavus* KCTC 1375; MIC = 20 μM against *C. albicans*.	[106]
Ib-AMP4 [111]	** E ** **WGRR** ** CC ** **GWGPGRRY** ** C ** **RRW** ** C **	+5
	IC_50_ = 3 (2.5)* μM against *B. cinerea* JHCC 8973; IC_50_ = 1.0 (2.5)* μM against *F. culmorum* IMI 180420; IC_50_ = 1.2 μM against *Neurospora crassa* FGSC 2489; IC_50_ = 13 μM against *Saccharomyces cerevisiae* BY4741; IC_50_ = 5 μM against *Pichia pastoris* GS115.	[111]
Ib-AMP1 [111]	** E ** **WGRR** ** CC ** **GWGPGRRY** ** C ** **VRW** ** C **	+4
	IC_50_ = 1.5 μM against *B. cinerea* JHCC 8973; IC_50_ = 1.4 μM against *F. culmorum* IMI 180420; IC_50_ = 0.5 μM against *N. crassa* FGSC 2489; IC_50_ = 15 μM against *S. cerevisiae* BY4741; IC_50_ = 16 μM against *P. pastoris* GS115;	[111]
	MIC = 16 μM against *E. coli* KCTC 1682; MIC > 32 μM against *Pseudomonas aeruginosa* KCTC 1637; MIC > 32 μM against *P. aeruginosa* (MDRPA) CCARM 2095; MIC > 32 μM against *Salmonella typhimurium* KCTC 1926; MIC = 16 μM against *B. subtilis* KCTC 3068; MIC = 16 μM against *Staphylococcus epidermidis* KCTC 1917; MIC = 16 μM against *S. aureus* KCTC 1621; MIC = 16 μM against *S. aureus* (MRSA) CCARM 3543.	[107]
Analog 1	** - ** **WGRR--GWGPGRRY-VRW** ** -NH_2_ **	nd
	MIC = 16 μM against *E. coli* KCTC 1682; MIC = 16 μM against *P. aeruginosa* KCTC 1637; MIC = 16 μM against *P. aeruginosa* (MDRPA) CCARM 2095; MIC = 4 μM against *S. typhimurium* KCTC 1926; MIC = 8 μM against *B. subtilis* KCTC 3068; MIC = 8 μM against *S. epidermidis* KCTC 1917; MIC = 4 μM against *S. aureus* KCTC 1621; MIC = 2 μM against *S. aureus* (MRSA) CCARM 3543.	[107]
Analog 2	** - ** **WGRR--GWGpGRRY-VRW** ** -NH_2_ **	nd
	MIC = 8 μM against *E. coli* KCTC 1682; MIC = 8 μM against *P. aeruginosa* KCTC 1637; MIC = 32 μM against *P. aeruginosa* (MDRPA) CCARM 2095; MIC = 4 μM against *S. typhimurium* KCTC 1926; MIC = 4 μM against *B. subtilis* KCTC 3068; MIC = 8 μM against *S. epidermidis* KCTC 1917; MIC = 4 μM against *S. aureus* KCTC 1621; MIC = 2 μM against *S. aureus* (MRSA) CCARM 3543.	[107]
Analog 3	** -- ** **WGRR--GWGaGRRY-VRW** ** -NH_2_ **	nd
	MIC = 16 μM against *E. coli* KCTC 1682; MIC = 16 μM against *P. aeruginosa* KCTC 1637; MIC = 16 μM against *P. aeruginosa* (MDRPA) CCARM 2095; MIC = 4 μM against *S. typhimurium* KCTC 1926; MIC = 4 μM against *B. subtilis* KCTC 3068; MIC = 8 μM against *S. epidermidis* KCTC 1917; MIC = 4 μM against *S. aureus* KCTC 1621; MIC = 2 μM against *S. aureus* (MRSA) CCARM 3543.	[107]
Analog 4	** -- ** **WGRR--GWGkGRRY-VRW** ** -NH_2_ **	nd
	MIC = 8 μM against *E. coli* KCTC 1682; MIC = 8 μM against *P. aeruginosa* KCTC 1637; MIC = 16 μM against *P. aeruginosa* (MDRPA) CCARM 2095; MIC = 4 μM against *S. typhimurium* KCTC 1926; MIC = 4 μM against *B. subtilis* KCTC 3068; MIC = 8 μM against *S. epidermidis* KCTC 1917; MIC = 4 μM against *S. aureus* KCTC 1621; MIC = 2 μM against *S. aureus* (MRSA) CCARM 3543.	[107]
MCE01	**<** ** E ** **WGRRBBGWGPGRRYBVRW** ** B **	nd
	IC_50_ = 1.5 μM against *B. cinerea* JHCC 8973; IC_50_ = 3 μM against *N. crassa* FGSC 2489; IC_50_ = 5 μM against *F. culmorum* IMI 180420; IC_50_ = 20 μM against *S. cerevisiae* BY4741; IC_50_ = 6 μM against *P. pastoris* GS115.	[111]
MCE02	** <E ** **WGRRBBGWGPGRRYBRRWB**	nd
	IC_50_ = 2 (4.5)* μM against *B. cinerea* JHCC 8973; IC_50_ = 3 μM against *N. crassa* FGSC 2489; IC_50_ = 5 (4.5)* μM against *F. culmorum* IMI 180420; IC_50_ = 20 μM against *S. cerevisiae* BY4741; IC_50_ = 4 μM against *P. pastoris* GS115.	[111]
MCD26	** <e ** **wgrrbbgwgpgrrybvrw** ** b **	nd
	IC_50_ = 0.5 μM against *B. cinerea* JHCC 8973; IC_50_ = 0.8 μM against *N. crassa* FGSC 2489; IC_50_ = 1.4 μM against *F. culmorum* IMI 180420; IC_50_ = 2 μM against *S. cerevisiae* BY4741; IC_50_ = 2 μM against *P. pastoris* GS115.	[111]
MCD30	** <e ** **wgrrbbgwgpgrrybrrwb**	nd
	IC_50_ = 1 μM against *B. cinerea* JHCC 8973; IC_50_ = 1.5 μM against *N. crassa* FGSC 2489; IC_50_ = 0.5 μM against *F. culmorum* IMI 180420; IC_50_ = 7.5 μM against *S. cerevisiae* BY4741; IC_50_ = 1 μM against *P. pastoris* GS115.	[111]
MCC02	**-** ** R ** **WGRRBBGWGPGRRYBRRWB**	nd
	IC_50_ = 3.5 μM against *B. cinerea* JHCC 8973.	[111]
MCC03	**<ERGRRBBGWGPGRRYBRRWB**	nd
	IC_50_ = 3.5 μM against *B. cinerea* JHCC 8973.	[111]
MCC04	**<EWRRRBBGWGPGRRYBRRWB**	nd
	IC_50_ = 3.0 μM against *B. cinerea* JHCC 8973.	[111]
MCC05	**<EWGRRRBGWGPGRRYBRRWB**	nd
	IC_50_ = 4.5 μM against *B. cinerea* JHCC 8973.	[111]
MCC06	**<EWGRRBRGWGPGRRYBRRWB**	nd
	IC_50_ = 3.0 μM against *B. cinerea* JHCC 8973.	[111]
MCC07	**<EWGRRBBRWGPGRRYBRRWB**	nd
	IC_50_ = 3.0 μM against *B. cinerea* JHCC 8973.	[111]
MCC08	**<EWGRRBBGRGPGRRYBRRWB**	nd
	IC_50_ = 2.5 μM against *B. cinerea* JHCC 8973.	[111]
MCC09	**<EWGRRBBGWRPGRRYBRRWB**	nd
	IC_50_ = 2.5 μM against *B. cinerea* JHCC 8973.	[111]
MCC10	**<EWGRRBBGWGRGRRYBRRWB**	nd
	IC_50_ = 2.5 μM against *B. cinerea* JHCC 8973.	[111]
MCC11	**<EWGRRBBGWGPRRRYBRRWB**	nd
	IC_50_ = 3.0 μM against *B. cinerea* JHCC 8973.	[111]
MCC12	**<EWGRRBBGWGPGRRRBRRWB**	nd
	IC_50_ = 3.0 μM against *B. cinerea* JHCC 8973.	[111]
MCC13	**<EWGRRBBGWGPGRRYRRRWB**	nd
	IC_50_ = 3.0 μM against *B. cinerea* JHCC 8973.	[111]
MCC14	**<EWGRRBBGWGPGRRYBRRRB**	nd
	IC_50_ = 4.0 μM against *B. cinerea* JHCC 8973.	[111]
MCC15	**<EWGRRBBGWGPGRRYBRRWR**	nd
	IC_50_ = 4.5 μM against *B. cinerea* JHCC 8973.	[111]
MCC16	**-** ** W ** **WGRRBBGWGPGRRYBRRWB**	nd
	IC_50_ = 6.5 μM against *F. culmorum* IMI 180420.	[111]
MCC17	**<EWWRRBBGWGPGRRYBRRWB**	nd
	IC_50_ = 3.0 μM against *F. culmorum* IMI 180420.	[111]
MCC18	**<EWGWRBBGWGPGRRYBRRWB**	nd
	IC_50_ = 3.0 μM against *F. culmorum* IMI 180420.	[111]
MCC19	**<EWGRWBBGWGPGRRYBRRWB**	nd
	IC_50_ = 3.0 μM against *F. culmorum* IMI 180420.	[111]
MCC21	**<EWGRRWBGWGPGRRYBRRWB**	nd
	IC_50_ = 4.5 μM against *F. culmorum* IMI 180420.	[111]
MCC22	**<EWWRRBWGWGPGRRYBRRWB**	nd
	IC_50_ = 2.5 μM against *F. culmorum* IMI 180420.	[111]
MCC23	**<EWWRRBBWWGPGRRYBRRWB**	nd
	IC_50_ = 3.0 μM against *F. culmorum* IMI 180420.	[111]
MCC24	**<EWWRRBBGWWPGRRYBRRWB**	nd
	IC_50_ = 3.0 μM against *F. culmorum* IMI 180420.	[111]
MCC25	**<EWWRRBBGWGWGRRYBRRWB**	nd
	IC_50_ = 3.0 μM against *F. culmorum* IMI 180420.	[111]
MCD17	**<EWWRRBBGWGPWRRYBRRWB**	nd
	IC_50_ = 3.5 μM against *F. culmorum* IMI 180420.	[111]
MCD18	**<EWWRRBBGWGPGWRYBRRWB**	nd
	IC_50_ = 3.5 μM against *F. culmorum* IMI 180420.	[111]
MCD19	**<EWWRRBBGWGPGRWYBRRWB**	nd
	IC_50_ = 3.5 μM against *F. culmorum* IMI 180420.	[111]
MCD21	**<EWWRRBBGWGPGRRWBRRWB**	nd
	IC_50_ = 3.0 μM against *F. culmorum* IMI 180420.	[111]
MCD22	**<EWWRRBBGWGPGRRYWRRWB**	nd
	IC_50_ = 3.0 μM against *F. culmorum* IMI 180420.	[111]
MCD23	**<EWWRRBBGWGPGRRYBWRWB**	nd
	IC_50_ = 3.0 μM against *F. culmorum* IMI 180420.	[111]
MCD24	**<EWWRRBBGWGPGRRYBRWWB**	nd
	IC_50_ = 2.0 μM against *F. culmorum* IMI 180420.	[111]
MCD25	**<EWWRRBBGWGPGRRYBRRWW**	nd
	IC_50_ = 2.0 μM against *F. culmorum* IMI 180420.	[111]
